# Regulatory interaction of BcWRKY33A and BcHSFA4A promotes salt tolerance in non-heading Chinese cabbage [*Brassica campestris* (syn. *Brassica rapa*) ssp. *chinensis*]

**DOI:** 10.1093/hr/uhac113

**Published:** 2022-05-17

**Authors:** Huiyu Wang, Zhubo Li, Haibo Ren, Changwei Zhang, Dong Xiao, Ying Li, Xilin Hou, Tongkun Liu

**Affiliations:** State Key Laboratory of Crop Genetics & Germplasm Enhancement, Key Laboratory of Biology and Genetic Improvement of Horticultural Crops (East China), Ministry of Agriculture and Rural Affairs of China, Engineering Research Center of Germplasm Enhancement and Utilization of Horticultural Crops, Ministry of Education of China, Nanjing Agricultural University, Nanjing 210095, China; State Key Laboratory of Crop Genetics & Germplasm Enhancement, Key Laboratory of Biology and Genetic Improvement of Horticultural Crops (East China), Ministry of Agriculture and Rural Affairs of China, Engineering Research Center of Germplasm Enhancement and Utilization of Horticultural Crops, Ministry of Education of China, Nanjing Agricultural University, Nanjing 210095, China; State Key Laboratory of Crop Genetics & Germplasm Enhancement, Key Laboratory of Biology and Genetic Improvement of Horticultural Crops (East China), Ministry of Agriculture and Rural Affairs of China, Engineering Research Center of Germplasm Enhancement and Utilization of Horticultural Crops, Ministry of Education of China, Nanjing Agricultural University, Nanjing 210095, China; State Key Laboratory of Crop Genetics & Germplasm Enhancement, Key Laboratory of Biology and Genetic Improvement of Horticultural Crops (East China), Ministry of Agriculture and Rural Affairs of China, Engineering Research Center of Germplasm Enhancement and Utilization of Horticultural Crops, Ministry of Education of China, Nanjing Agricultural University, Nanjing 210095, China; State Key Laboratory of Crop Genetics & Germplasm Enhancement, Key Laboratory of Biology and Genetic Improvement of Horticultural Crops (East China), Ministry of Agriculture and Rural Affairs of China, Engineering Research Center of Germplasm Enhancement and Utilization of Horticultural Crops, Ministry of Education of China, Nanjing Agricultural University, Nanjing 210095, China; State Key Laboratory of Crop Genetics & Germplasm Enhancement, Key Laboratory of Biology and Genetic Improvement of Horticultural Crops (East China), Ministry of Agriculture and Rural Affairs of China, Engineering Research Center of Germplasm Enhancement and Utilization of Horticultural Crops, Ministry of Education of China, Nanjing Agricultural University, Nanjing 210095, China; Nanjing Suman Plasma Engineering Research Institute, Nanjing Agricultural University, Nanjing 210095, China; State Key Laboratory of Crop Genetics & Germplasm Enhancement, Key Laboratory of Biology and Genetic Improvement of Horticultural Crops (East China), Ministry of Agriculture and Rural Affairs of China, Engineering Research Center of Germplasm Enhancement and Utilization of Horticultural Crops, Ministry of Education of China, Nanjing Agricultural University, Nanjing 210095, China; Nanjing Suman Plasma Engineering Research Institute, Nanjing Agricultural University, Nanjing 210095, China; State Key Laboratory of Crop Genetics & Germplasm Enhancement, Key Laboratory of Biology and Genetic Improvement of Horticultural Crops (East China), Ministry of Agriculture and Rural Affairs of China, Engineering Research Center of Germplasm Enhancement and Utilization of Horticultural Crops, Ministry of Education of China, Nanjing Agricultural University, Nanjing 210095, China

## Abstract

Salinity is a universal environmental stress that causes yield reduction in plants. WRKY33, which has been extensively studied in plant defense against necrotrophic pathogens, has recently been found to be important in salt-responsive pathways. However, the underlying molecular mechanisms controlling the involvement of *WRKY33* in salt tolerance have not been fully characterized. Here, we explored the function of BcWRKY33A in non-heading Chinese cabbage (NHCC). Under salt stress, *BcWRKY33A* expression is significantly induced in roots. As a nuclear protein, BcWRKY33A has strong transcriptional activation activity. Overexpression of *BcWRKY33A* confers salt tolerance in *Arabidopsis*, whereas silencing of *BcWRKY33A* causes salt sensitivity in NHCC. Furthermore, BcHSFA4A, a protein that interacts with BcWRKY33A, could directly bind to the HSE motif within the promoters of *BcZAT12* and *BcHSP17.6A*, which are involved in the plant response to salt stress. Finally, we found that BcWRKY33A could enhance the transcriptional activity of BcHSFA4A and affect its downstream genes (e.g. *BcZAT12* and *BcHSP17.6A*), and co-overexpression of *BcWRKY33A* and *BcHSFA4A* could promote the expression of salt-related genes, suggesting that the regulatory interaction between BcWRKY33A and BcHSFA4A improves salt tolerance in plants. Overall, our results provide insight into the molecular framework of the BcWRKY33A-BcHSFA4A signaling pathway, which also aids in our understanding of the molecular mechanism of salt tolerance in plants.

## Introduction

Salinity is a common environmental stress that threatens crop development and production [1]. Under global warming and severe environmental challenges, increasing soil salinization has led to a gradual decrease in the cultivated land area worldwide [2]. During all stages of plant growth, salt stress often leads to osmotic stress, resulting in excess reactive oxygen species (ROS) production, impaired photosynthesis, growth suppression, and even plant death [3]. In previous studies, many transcription factors (TFs) were found to be involved in plant salt tolerance, such as MYB, bZIP, NAC, WRKY, and HSF [4]. For instance, under salt stress, high levels of *AtMYB20* enhance *Arabidopsis* salt tolerance by repressing *PP2C* expression [5]. In grapevines (*Vitis vinifera*), MYB108A-mediated ethylene biosynthesis could be promoted by melatonin, thus enhancing plant salt tolerance [6]. Additionally, *AtbZIP17* is highly expressed under salt stress and improves plant salt tolerance by affecting the expression levels of its target genes, such as *AtRD29A* and *AtRD20* [7]. In wheat, TaNAC29 can increase plant antioxidant enzyme activity and reduce H_2_O_2_ accumulation and membrane damage under salt stress [8]. MlNAC9, an NAC transcription factor in *Miscanthus*, can confer salt tolerance to *Arabidopsis* plants via an abscisic acid (ABA)-dependent pathway [9]. In apple (*Malus* × *domestica* Borkh.), the NAC transcription factor MdSND1 shows various expression patterns under salt stress, mannitol or ABA treatment, and *MdSND1*-overexpressing apple plants exhibit salt and osmotic tolerance compared with wild-type (WT) plants [10]. Therefore, further in-depth research on the interaction mechanism or regulatory relationship among salt-responsive TFs may contribute to understanding of the salt-responsive mechanism of plants.

The WRKY family includes largely conserved TFs that are responsive to multiple abiotic stresses in many plants [11–14]. Under high-salt conditions, *TaWRKY44* in wheat was induced significantly, and tobacco overexpressing *TaWRKY44* showed enhanced salt tolerance [15]. Similarly, overexpression of *TaWRKY93*, another salt-inducible gene, in *Arabidopsis* resulted in longer primary roots or more lateral roots, a higher proline content, and an increased survival rate relative to the WT under salt stress conditions [16]. Moreover, compared with WT, the overexpression of *GhWRKY34* in *Arabidopsis* results in a higher germination rate and chlorophyll content, as well as a longer root length, which suggests that transgenic *Arabidopsis* has stronger salt tolerance [17]. However, genetically modified tobacco overexpressing *GhWRKY68* exhibits higher salt sensitivity than WT tobacco [18]. Hence, many members of the WRKY family perform functions in the processes that plants use to respond to salt stress. Previous studies have shown that WRKY33 could mediate the response to necrotrophic fungal pathogens [19, 20], and we also provided evidence that sigma factor binding proteins (SIBs) interact with WRKY33 and activate it during the defense response to necrotrophic pathogens, thereby improving the disease resistance of plants [21]. However, in recent studies, *AtWRKY33* has also been shown to respond to salt stress [22–24]. Overexpression of *AtWRKY33* in *Arabidopsis* can result in enhanced tolerance to salt stress [22]. Furthermore, other studies found that AtWRKY33 can regulate root apoplastic barrier formation by directly binding to the *AtCYP94B1* promoter, conferring enhanced salt tolerance to plants [23]. Additionally, AtWRKY33 was identified as a direct regulator of *AtKUP2*, whose overexpression lines showed increased root and stem length and higher germination and survival rates than the WT [24]. All these studies suggest that AtWRKY33 may act as a direct or indirect regulatory factor to coordinate or control downstream salt stress-related genes and affect plant salt tolerance; however, the molecular mechanism by which WRKY33 participates in the process of plant resistance to salt stress needs to be further investigated. Hence, WRKY33, as a transcription factor with multiple resistance ability (biotic and abiotic stress), attracted our interest.

The heat shock transcription factor family (HSFs) is also generally involved in plant responses to stressful environments, such as extreme temperatures, poor soil environments, osmotic stress, and pathogen attack [25]. The ectopic overexpression of *SIHsfA3* from tomato (*Solanum lycopersicum*) in *Arabidopsis* increased the high-temperature tolerance but decreased the salt tolerance of transgenic plants [26]. In maize (*Zea mays*), *ZmHsf08* regulates stress-related genes to cope with high-salt and drought conditions [27]. In *Arabidopsis*, AtHSFA7b is a *trans*-activator that can bind to the E-box motif in the promoters of *bHLH*, *NAC061*, *NAC036*, *NAC090*, *WRKY38*, and *ZFP2* genes, regulate their expression and mediate serial physiological programs to increase plant salt tolerance [28]. Likewise, HSFA4A improves the expression of downstream defense TFs (ZnF, MYB, WRKY, etc.) to confer plant salt tolerance [29]. Although both WRKY33 and HSFA4A are involved in the process of salt tolerance in plants, their regulatory mechanism is unclear.

Non-heading Chinese cabbage [NHCC, *Brassica campestris* (syn. *Brassica rapa*) ssp. *chinensis*], with high yield and nutritional value, remains popular among consumers [30]. However, its cultivation usually faces risks from high salinity. In the seed germination stage, salt stress results in delayed and non-uniform seed germination [31]. In the growth stage, salt stress will lead to high osmotic pressure in the soil, which limits the absorptive capacity and normal growth of the roots, resulting in plant wilting and leaf yellowing. Therefore, the improvement of salt tolerance in NHCC is important for its production. Here, we explored the molecular mechanism of BcWRKY33A involvement in salt tolerance and found that BcWRKY33A could interact with BcHSFA4A at the protein level. This interaction could enhance the transcriptional activation activity of BcHSFA4A to promote the expression of downstream genes (*BcZAT12* and *BcHSP17.6A*) in NHCC. Moreover, the transgenic line co-overexpressing *BcWRKY33A* and *BcHSFA4A* exhibited increased salt-related gene expression and plant salt tolerance. Hence, our results suggest that coordinated regulation of BcWRKY33A and BcHSFA4A improves the salt tolerance of NHCC.

## Results

### *BcWRKY33* could be induced by salt stress in NHCC

**Figure 1:**
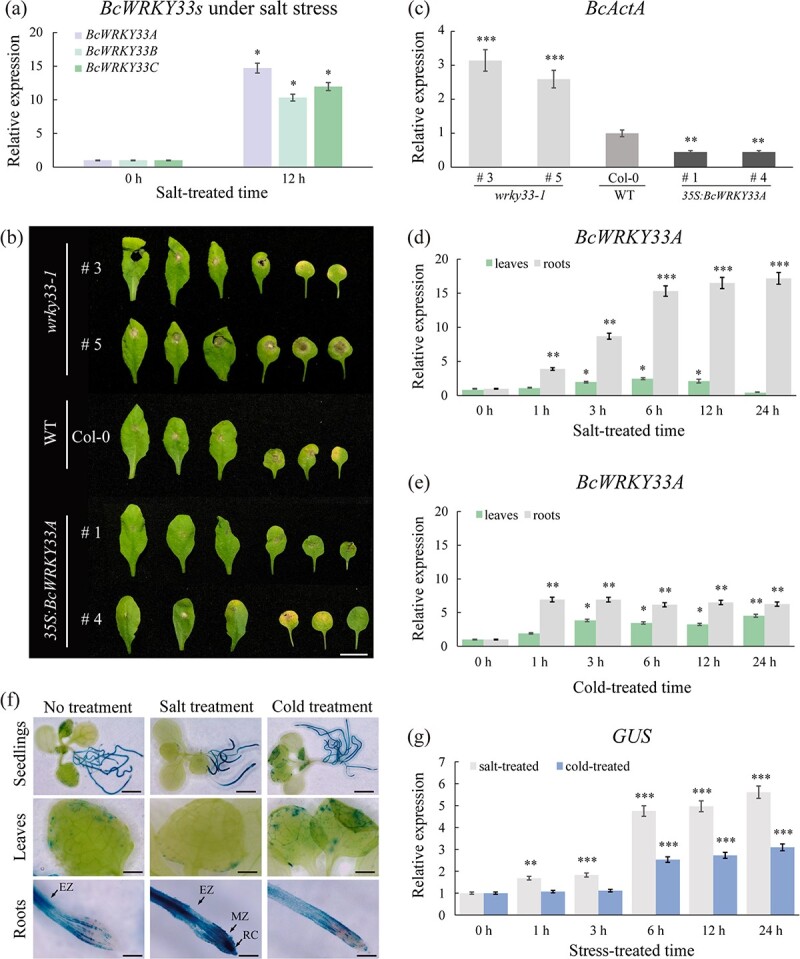
Identification of BcWRKY33A and its expression pattern under stress conditions. **a** Relative expression of *BcWRKY33s* in salt-treated NHCC ‘Suzhouqing’ plants at 0 and 12 hours. **b** Phenotypes of WT (Col-0), *wrky33-1* (SALK_006603, #3, #5), and *35S:BcWRKY33A* (#1, #4) lines after inoculation with *B. cinerea*. Disease symptoms were recorded 4 days post-*Botrytis* inoculation (dpi). Scale bar = 1 cm. **c** Expression of the *Botrytis ActinA* (*BcActA*) gene in spray-inoculated plants at 4 dpi. **d**, **e** Expression of *BcWRKY33A* in leaves and roots of salt-treated (**d**) and cold-treated (**e**) NHCC ‘Suzhouqing’ plants at different times. **f** GUS staining of *BcWRKY33A:GUS* transgenic *Arabidopsis* lines in which *GUS* gene expression was driven by the *BcWRKY33A* promoter under mock, salt (100 mM NaCl, 12 hours), or cold (4°C, 12 hours) treatment. Scale bar, top to bottom, 2.5 mm, 1 mm, and 500 μm, respectively. EZ, elongation zone; MZ, meristem zone; RC, root cap. **g** Expression of *GUS* after salt or cold treatment in *BcWRKY33A:GUS* seedlings from (**f**). All data are averages of three independent experiments, and error bars represent the standard error of the mean. ^*^P < .05, ^**^P < .01, ^***^P < .001 (Student’s *t*-test).

After divergence from *Arabidopsis thaliana*, a whole-genome triplication event occurred in *Brassica* species [30]. Three homologs of *Arabidopsis* WRKY33 were found in the NHCC genome and named BcWRKY33A, BcWRKY33B, and BcWRKY33C, as they share similar domains and high amino acid identities (Supplementary Data Fig. S1a and b). Phylogenetic analysis showed that the BcWRKY33A (BraC04g029190.1), BcWRKY33B (BraC03g020440.1) and BcWRKY33C (BraC05g007540.1) proteins were closely related to WRKY33 from *A. thaliana*, *B. rapa* subsp. *oleifera* and *Raphanus sativus* (Supplementary Data Fig. S1c). This result suggested that BcWRKY33A, which shows the highest homology with AtWRKY33, may share a similar function. Moreover, to initially investigate the response of *BcWRKY33s* to salt stress, their expression levels in salt-treated seedlings of NHCC cultivar ‘Suzhouqing’ were detected by quantitative real-time PCR (qPCR). We found that *BcWRKY33A* had the highest expression level among the three homologs (Fig. 1a). Here, BcWRKY33A, the closest homolog of AtWRKY33, exhibited the most obvious degree of induction under salt stress and was selected for the next study.

Similar to AtWRKY33, BcWRKY33A was also involved in resistance to *Botrytis cinerea* (*B. cinerea*) (Fig. 1b and c; Supplementary Data Fig. S2a–c) [32]. When faced with salt stress, *BcWRKY33A* could be rapidly induced in roots, where it continuously increased by 3- to 18-fold, stabilized and remained at a high level. In leaves, it increased within 12 hours and then decreased back to the initial level at 24 hours (Fig. 1d), suggesting that BcWRKY33A mainly functions in roots rather than leaves and participates in the salt response pathway. In addition, under 4°C cold treatment, *BcWRKY33A* responded quickly, and its expression also increased by 2- to 10-fold in both leaves and roots (Fig. 1e).

To examine the spatial expression pattern of BcWRKY33A, the *BcWRKY33A*-promoter-controlled *GUS* (β-glucuronidase) reporter (*BcWRKY33A:GUS*) was constructed and transformed into *Arabidopsis*. Under normal conditions, the BcWRKY33A:GUS signal was detected in roots (mainly in the elongation zone) and leaves. After salt treatment for 12 hours, the BcWRKY33A:GUS signal was sharply induced and markedly enriched in the roots, especially in the elongation zone, meristem zone, and root cap, but it was barely detected in leaves when compared with the untreated plants, suggesting that BcWRKY33A played an important role in root growth (Fig. 1f). In addition, under 4°C cold treatment, BcWRKY33A:GUS activity increased slightly in roots and leaves (Fig. 1f). Then, 12-day-old transgenic seedlings harboring *BcWRKY33A:GUS* were treated with salt (100 mM NaCl) or cold (4°C) for various periods of time. The qRT–PCR results showed that *BcWRKY33A:GUS* responded to stress conditions (e.g. salt and cold); however, its response was more rapid and obvious in salt-treated plants than in cold-treated plants (Fig. 1g). Thus, we suggest that *BcWRKY33A* is induced and functions mainly in the roots under salt stress.

### BcWRKY33A is a nuclear protein with strong transcriptional activation activity

**Figure 2:**
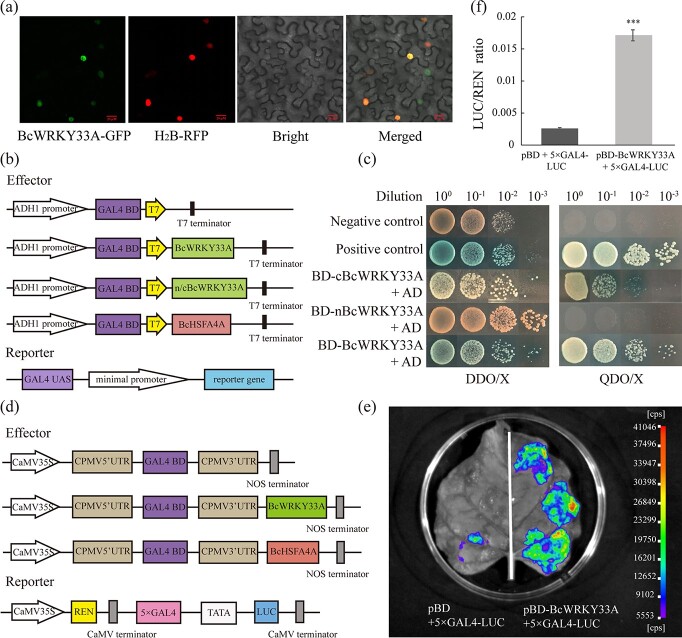
Subcellular localization and transcriptional activation activity of BcWRKY33A. **a** BcWRKY33A located in the nucleus was analyzed by confocal microscopy. BcWRKY33A-GFP and the nuclear marker H_2_B-RFP overlapped in the nucleus. Scale bars = 20 μm. **b** Illustration of reporter and effector constructs used in the Y2H assay. **c** Transcriptional activation activity of BcWRKY33A, nBcWRKY33A, and cBcWRKY33A in yeast. Positive control, BD-53 + AD-T; negative control, pGBKT7 + pGADT7. DDO/X and QDO/X represent SD/−Trp/−Leu and SD/−Trp/−His/−Leu/−Ade media supplemented with 4 mg mL^−1^ X-α-gal, respectively. **d** Illustration of reporter and effector constructs used for transcriptional activation activity analysis of BcWRKY33A. **e** Imaging of LUC activity in tobacco leaves injected with pBD + 5 × GAL4-LUC (negative control) and pBD-BcWRKY33A + 5 × GAL4-LUC. **f** LUC activities were measured as the LUC/REN ratio in tobacco leaves. All data are averages of three independent experiments, and error bars represent the standard error of the mean. ^***^P < .001 (Student’s *t*-test).

Since *Arabidopsis* WRKY33 has been identified as a transcription factor [20], we speculated that BcWRKY33A is also a TF located in the nucleus. Thus, we constructed a recombinant BcWRKY33A-GFP vector and examined its location via transient expression in tobacco. The histone H_2_B-RFP fusion protein was co-injected as a nuclear marker [33]. As shown in Fig. 2a, BcWRKY33A-GFP and H_2_B-RFP overlapped in the nucleus, suggesting that the BcWRKY33A protein was located in the cell nucleus.

To further understand the function of *BcWRKY33A*, the transcriptional activity of BcWRKY33A was examined in a yeast system. Similar to the positive control, the Y2H Gold yeast strain containing BD-BcWRKY33A and the activation domain (AD) grew on QDO/X medium, which indicates that BcWRKY33A has strong transcriptional activation activity (Fig. 2b and c). To further determine its activation region in detail, the full-length BcWRKY33A protein was truncated into nBcWRKY33A and cBcWRKY33A (Supplementary Data Fig. S1a). The results indicated that cBcWRKY33A had strong transcriptional activation activity, although it was slightly weaker than that of full-length BcWRKY33A (Fig. 2c). Moreover, the transcriptional activity of BcWRKY33A *in vivo* was also verified by a dual-luciferase (LUC) assay [34]. Compared with the control, pBD-BcWRKY33A remarkably promoted the luciferase (LUC) activity and LUC/REN ratio (Fig. 2d–f), supporting the results in yeast. Together, the BcWRKY33A protein, which is located in the nucleus, has strong transcriptional activation activity.

### Overexpression of *BcWRKY33A* enhances the salt tolerance of *A. thaliana*

**Figure 3:**
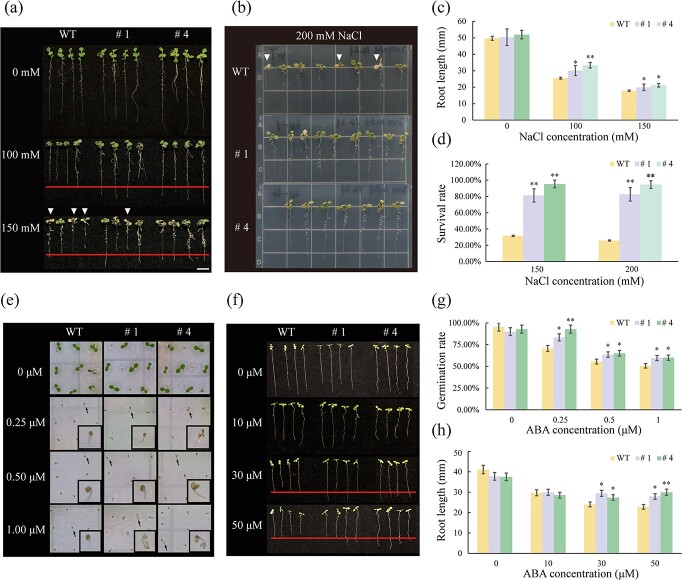
Heterologous expression of *BcWRKY33A* increases salt tolerance and decreases ABA sensitivity in *A. thaliana*. **a**–**d** Phenotype (**a**, **b**), root length (**c**), and survival rate (**d**) of WT and *35S:BcWRKY33A* transgenic *Arabidopsis* lines (#1, #4) under salt stress. The red line represents the longest root length of WT. The white triangles represent dead seedlings under salt stress. **e**, **f** Germination (**e**) and root length (**f**) phenotypes of WT and *35S:BcWRKY33A* transgenic *Arabidopsis* lines (#1, #4) under mock or ABA treatment. The arrows in (**e**) indicate the seedlings that were magnified and observed. **g, h** Germination rate (**g**) and root length (**h**) analysis of WT and *35S:BcWRKY33A* transgenic *Arabidopsis* lines (#1, #4) under mock or ABA treatment. All data are averages of three independent experiments, and error bars represent the standard error of the mean. ^*^P < .05, ^**^P < .01 (Student’s *t*-test).

Based on the significant induction of *BcWRKY33A* by salt treatment, we assumed that *BcWRKY33* could regulate the salt tolerance of plants. Then, we analyzed the phenotype of transgenic *35S:BcWRKY33A-GFP Arabidopsis* lines under salt stress (Supplementary Data Fig. S2c). Under normal growth conditions, the transgenic *35S:BcWRKY33A-GFP* (#1, #4) and WT plants had similar phenotypes (Fig. 3a). However, when seedlings were subjected to salt treatment for 7 days, the root growth and survival rate exhibited significant differences (Fig. 3a and b). Compared with the WT, the transgenic lines had longer root lengths and more lateral roots under NaCl treatment (100 and 150 mM) (Fig. 3a and c), which indicates that the transgenic plants may have a better ability to absorb water and nutrients. Moreover, the transgenic lines had a higher survival rate, and this phenomenon was more pronounced at higher salt concentrations (150 and 200 mM) (Fig. 3a, b and d). Therefore, overexpression of *BcWRKY33A* in *Arabidopsis* leads to more lateral roots, longer root length and higher survival rate than WT, which could enhance the salt tolerance of transgenic *Arabidopsis*.

ABA, which regulates salt stress-responsive gene expression, is the central regulator of salt tolerance, and the application of ABA could mimic the effects of various stresses on plants [35]. Moreover, ABA plays a crucial role in the processes of seed germination and root development [36, 37]. To study whether *BcWRKY33A* responds to ABA, we examined the seed germination and root growth of WT and *35S:BcWRKY33A-GFP* transgenic lines under various ABA concentrations. As predicted, with increased ABA concentrations, the germination of transgenic seeds at 5 days was less sensitive to ABA than that of WT plants (Fig. 3e and f). Even if the ABA concentration was increased to 1 μM, the germination rate of transgenic seeds remained at 59.57 and 60.21%, higher than the 50.54% found for WT seeds (Fig. 3e and f). Moreover, under the higher concentrations of ABA (30–50 μM) for 7 days, the root lengths of transgenic seedlings (#1, #4) were significantly longer than those of WT (Fig. 3f and h). Thus, we suggest that *BcWRKY33A* enhances plant salt tolerance by promoting lateral root growth and primary root elongation and increasing the survival rate and germination rate.

### Silencing of *BcWRKY33A* causes salt sensitivity in NHCC plants

**Figure 4:**
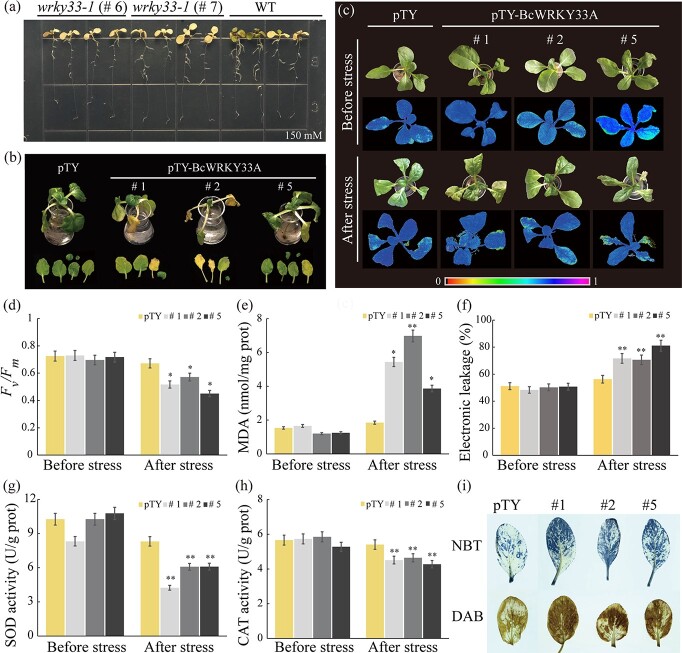
Silencing of *BcWRKY33A* impairs salt tolerance in NHCC. **a** Phenotype of WT and *wrky33-1* mutants (#6, #7) under salt treatment (150 mM NaCl, 7 days). **b** Phenotype of pTY and silenced lines (#1, #2, #5) before and after salt treatment (100 mM NaCl, 8 days). **c**, **d** Chlorophyll fluorescence imaging (**c**) and *F*_v_*/F*_m_ ratios (**d**) in the pTY and silenced lines (#1, #2, #5), measured before and after salt treatment (100 mM NaCl, 5 days). **e**, **f** MDA content and electrolyte leakage in pTY and silenced lines (#1, #2, #5) before and after salt treatment. **g**, **h** SOD and CAT activity in pTY and silenced lines (#1, #2, #5) before and after salt treatment. **i** Histochemical staining with DAB and NBT for visualization of H_2_O_2_ and O2^.−^, respectively, in pTY and silenced lines (#1, #2, #5) after salt treatment. All data are averages of three independent experiments, and error bars represent the standard error of the mean. ^*^P < .05, ^**^P < .01 (Student’s *t*-test).

In *Arabidopsis*, the primary root elongation of *wrky33-1* mutants was inhibited by salt stress [20]. However, it is unclear whether the lateral root phenotype of *wrky33-1* mutants is affected by salt stress. Phenotypic analyses revealed that *wrky33-1* plants always showed fewer lateral roots than WT plants under salt treatment (Fig. 4a), which is consistent with the above results showing that BcWRKY33A promotes lateral root development and enhances plant salt tolerance (Fig. 3a and b).

To further confirm the role of BcWRKY33A in NHCC, we obtained *BcWRKY33A*-silenced lines (pTY-BcWRKY33A, #1, #2, and #5) of the NHCC cultivar ‘Suzhouqing’ that were produced by virus-induced gene silencing (VIGS). Since the silencing efficiency is most obvious in the leaves [38], we selected the leaves of silenced plants for further analysis (Fig. 4). After infection for 10 days, the mosaic phenotypes of the leaves and the expression of *BcWRKY33s* were examined (Supplementary Data Fig. S2d–g). Lines #1, #2, and #5 showed phenotypes similar to those of the pTY control before the stress treatment. However, lines #1, #2, and #5 showed more curled and yellowed leaves than the pTY control under salt stress conditions (Fig. 4b). Additionally, lines #1, #2, and #5 displayed decreased chlorophyll fluorescence and photosynthetic efficiency (*F*_v_/*F*_m_) relative to pTY plants (Fig. 4c and d). Thus, these results suggested that *BcWRKY33A*-silenced lines exhibited salt sensitivity and impaired photosynthetic capacity compared with the control under salt stress.

The malondialdehyde (MDA) content and electrolyte leakage rate could reflect membrane damage in stressed plants [39]. Under salt stress conditions, *BcWRKY33A*-silenced plants exhibited significantly higher levels of MDA and electrolyte leakage (Fig. 4e and f), implying more severe membrane injuries. Similarly, quantitative measurement and histochemical staining showed that lines #1, #2, and #5 accumulated more ROS than the control (Fig. 4g–i). Overall, the silencing of *BcWRKY33A* in NHCC plants led to increased salt sensitivity, further illustrating that an abundance of BcWRKY33A positively affects the salt tolerance of plants.

### BcHSFA4A interacts with BcWRKY33A and has weak transcriptional activation activity

**Figure 5:**
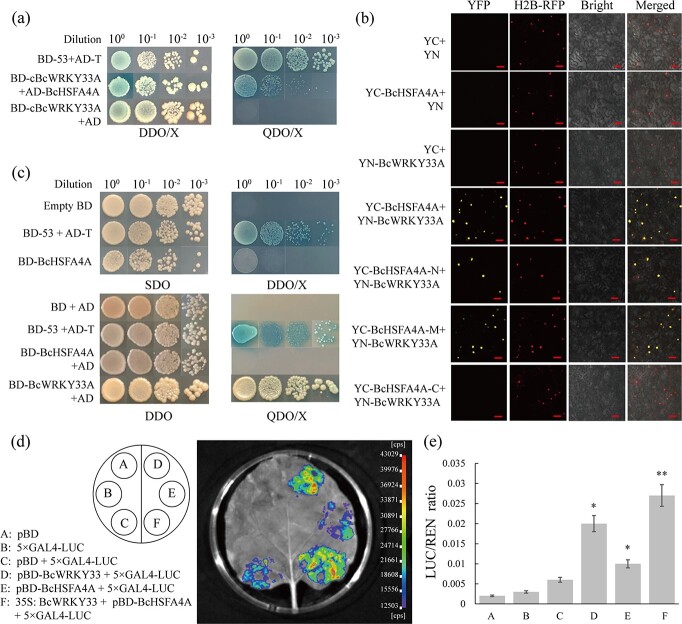
BcHSFA4A interacts with BcWRKY33A and has weak transcriptional activation activity. **a** Interaction between BcWRKY33A and BcHSFA4A in yeast. Positive control, BD-53 + AD-T; negative control, BD-cBcWRKY33A + AD. DDO/X and QDO/X represent SD/−Trp/−Leu and SD/−Trp/−His/−Leu/−Ade medium supplemented with 4 mg mL^−1^ X-α-gal, respectively. **b** Interaction between BcWRKY33A and BcHSFA4A in tobacco leaf epidermal cells. YFP, YFP fluorescence; H_2_B-RFP, nuclear marker, RFP fluorescence; Bright, bright-field image; Merged, merge of YFP, H_2_B-RFP, and Bright. Scale bars = 50 μm. **c** Transcriptional activation activity of BcHSFA4A and BcWRKY33A in yeast. SDO and DDO represent SD/−Trp and SD/−Trp/−His medium, respectively. DDO/X and QDO/X, as described in (**a**). **d** Imaging of LUC activity in tobacco leaves injected with different constructs. **e** LUC activity in tobacco leaves. A–F correspond to the labels in (**d**). All data are averages of three independent experiments, and error bars represent the standard error of the mean. ^*^P < .05, ^**^P < .01 (Student’s *t*-test ).

To further explore the regulatory mechanism of *BcWRKY33A*, yeast two-hybrid (Y2H) experiments were performed to identify proteins related to salt tolerance in NHCC. Interestingly, BraC01g010360.1, the homolog of *Arabidopsis HSFA4A*, which participates in salt and heat stress [29, 40, 41], was identified and designated *BcHSFA4A*. Later, the interaction between cBcWRKY33A (containing two conserved WRKY domains) and BcHSFA4A was confirmed in the Y2H assay (Fig. 5a), which suggested that BcWRKY33A interacted with BcHSFA4A via its WRKY domains. The bimolecular fluorescence complementation (BiFC) test was used to verify the Y2H results (Fig. 5b, upper four rows) and suggested that BcHSFA4A and BcWRKY33A directly interacted and were located in the nucleus. To further confirm the interaction region, BcHSFA4A was divided into three parts according to its protein domains (Supplementary Data Fig. S3). We found that BcWRKY33A could interact with the BcHSFA4A-N and BcHSFA4A-M fragments (Fig. 5b, lower three rows). Therefore, these results suggested that BcWRKY33A interacted with BcHSFA4A-N and BcHSFA4A-M via its WRKY domains and was located in the cell nucleus. To further understand the relationship between these two proteins, the expression levels of *HSFA4A* and *BcHSFA4A* were examined in *BcWRKY33A*-overexpressing and *BcWRKY33A*-silenced lines. Compared with the expression pattern of *BcWRKY33A* (Supplementary Data Fig. S2c and d), the expression level of *HSFA4A*/*BcHSFA4A* remained stable in WT, BcWRKY33A-overexpressing, and BcWRKY33A-silenced lines (Supplementary Data Fig. S3b and c), indicating that *HSFA4A*/*BcHSFA4A* was not affected by *BcWRKY33* at the transcript level.

**Figure 6:**
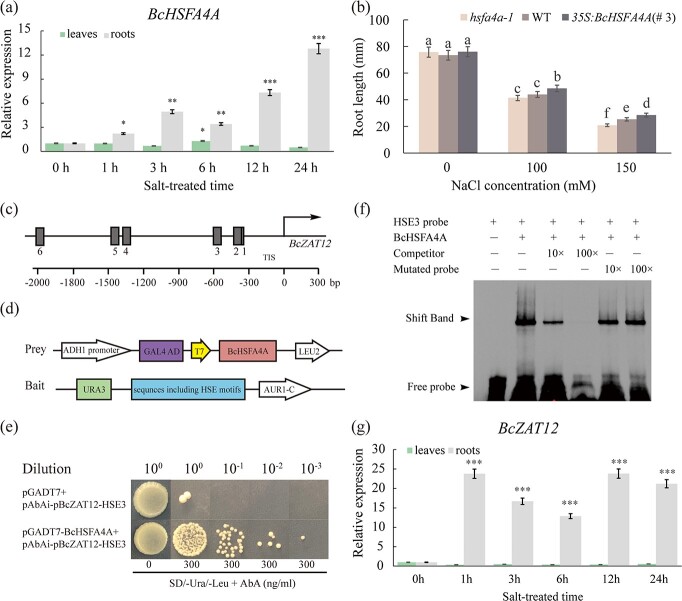
BcHSFA4A directly binds to the HSE motif in the promoter of *BcZAT12.***a** Relative expression of *BcHSFA4A* in salt-treated NHCC ‘Suzhouqing’ plants. **b** Root length of *hsfa4a-1*, WT, and *35S:BcHSFA4A* under mock or salt treatment for 10 days. Different letters indicate statistically significant differences at the level of *P* < .05. **c** Distribution of HSE motifs in the promoter of *BcZAT12*. HSE motifs are indicated by gray boxes. The arrow indicates the translation initiation site (TIS). **d** Illustration of the bait/prey constructs used in the Y1H assay. **e** Growth of yeast cells co-transformed with different constructs on selective medium with 0 or 300 ng mL^−1^ aureobasidin A (AbA). Empty pGADT7 was used as the negative control. **f** EMSA was used to detect the direct binding of BcHSFA4A to the HSE motif in the *BcZAT12* promoter. EMSA of the biotin-labeled oligonucleotide derived from the putative HSE4 binding site of the *BcZAT12* promoter in the presence or absence of a cold competitor and mutated probe. Purified BcHSFA4A protein (4 μg) was incubated with 50 nM biotin-labeled probes. For the competition test, cold competitor and mutated probes at 10- (10×) or 100-fold (100×) concentrations were added during the experiment. Presence (+) or absence (−) of the components is shown at the top. **g** Relative expression of *BcZAT12* in salt-treated NHCC ‘Suzhouqing’ plants. All data are averages of three independent experiments, and error bars represent the standard error of the mean. ^*^P < .05, ^**^P < .01, ^***^P < .001 (Student’s *t*-test ).

In the above data, we found that BcWRKY33A has strong transcriptional activation activity (Fig. 2c and e). Due to its interaction with BcWRKY33A, the transcriptional activation activity of BcHSFA4A was also examined. Yeast cells containing BD-BcHSFA4A could grow on DDO/X medium, albeit only slightly (Figs 2b and 5c). However, compared with the strong transcriptional activation activity of BcWRKY33A, yeast cells containing BD-BcHSFA4A and AD were unable to grow on QDO/X medium, implying that BcHSFA4A may have weak transcriptional activation activity (Figs 2c and 5c). Likewise, co-transformation of the effector pBD-BcHSFA4A and the reporter 5 × GAL4:LUC showed an increased LUC/REN ratio compared with the negative control but a decreased LUC/REN ratio compared with BD-BcWRKY33A (Fig. 5d and e), which is in accordance with the results in yeast. Together, these results suggested that BcHSFA4A, an interacting protein of BcWRKY33A, has weak transcriptional activation activity.

### BcHSFA4A participates in the response to salt stress by directly binding to the *BcZAT12* and *BcHSP17.6A* promoters

The idea that HSFA4A confers salt tolerance was developed in *Arabidopsis* [29, 40, 41]. However, the biological functions, especially the salt tolerance, of *BcHSFA4A* in NHCC are unknown. Under salt stress, *BcHSFA4A* was strongly induced in roots but almost unchanged in leaves (Fig. 6a), where its expression pattern was similar to that of *BcWRKY33A* (Fig. 1d). To further understand the biological functions of *BcHSFA4A* under salt stress, stable *35S:BcHSFA4A Arabidopsis* lines (#1, #2, and #3) were established, and line #3 was selected for testing (Supplementary Data Fig. S2h and i). Compared with the *hsfa4a-1* mutant and WT, *35S:BcHSFA4A* (#3) exhibited longer root lengths under different concentrations of NaCl (Fig. 6b; Supplementary Data Figs S2h and i and S4), indicating the positive role of *BcHSFA4A* in plant salt tolerance. However, the molecular regulatory mechanism of *BcHSFA4A* under salt stress conditions has not been characterized.

Previously, HSFA4A was speculated to bind to the promoters of *ZAT12* and *HSP17.6A* during the response to salt stress [40], but there was no experimental evidence for this finding. Here, HSE motifs were found in the promoters of *BcZAT12* (*BraC10g018540.1*) and *BcHSP17.6A* (*BraC03g005280.1*) (Fig. 6c; Supplementary Data Fig. S5a). For *BcZAT12*, its 2-kb promoter region was divided into four parts: HSE12 (containing HSE motifs 1 and 2, from −413 to −1 bp), HSE3 (containing HSE motif 3, from −588 to −414 bp), HSE45 (containing HSE motifs 4 and 5, from −1528 to −589 bp), and HSE6 (containing HSE motif 6, from −2000 to −1529 bp). The Y1H results revealed that BcHSFA4A could directly bind to HSE3 (Fig. 6d and e) but not to HSE12, HSE45, or HSE6 (not shown). Then, an electrophoretic mobility shift assay (EMSA) confirmed the direct binding of BcHSFA4A to HSE motif 3 (HSE3 probe) within the promoter of *BcZAT12* (Fig. 6f). In addition, we analyzed *BcZAT12* expression under salt stress, and the results showed that it increased rapidly within 1 hour, then decreased slightly as the time under salt treatment increased to 3–6 hours, and finally stabilized and remained at a high level at 12 hours in root tissue. At the same time, this expression level was almost unchanged in leaves (Fig. 6g), indicating that *BcZAT12* is strictly regulated in the roots under salt stress and implying that *BcZAT12* is positively involved in plant salt tolerance.

**Figure 7:**
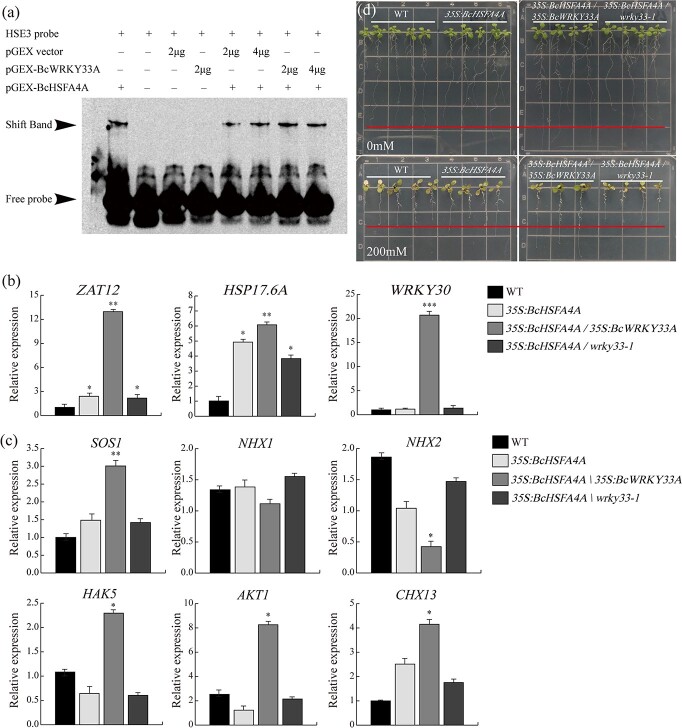
The BcWRKY33A-BcHSFA4A module enhances salt tolerance in plants. **a** EMSA was used to detect the effect of BcWRKY33A on binding ability between BcHSFA4A and the HSE motif in the *BcZAT12* promoter. Presence (+) or absence (−) of the components is shown at the top. **b** Expression of *ZAT12*, *HSP17.6A* and *WRKY30* in WT, *35S:BcHSFA4A*, *35S:BcHSFA4A/35S:BcWRKY33A*, and *35S:BcHSFA4A/wrky33-1* lines. **c** Expression levels of salt-related genes (*SOS1*, *NHX1*, *NHX2*, *HAK5*, *AKT1*, *CHX13*) in WT, *35S:BcHSFA4A*, *35S:BcHSFA4A/35S:BcWRKY33A*, and *35S:BcHSFA4A/wrky33-1* lines. **d** Phenotypes of WT, *35S:BcHSFA4A*, *35S:BcHSFA4A/35S:BcWRKY33A*, and *35S:BcHSFA4A/wrky33-1* lines under normal conditions and salt treatment. The red line represents the longest root length in the WT. All data are averages of three independent experiments, and error bars represent the standard error of the mean. ^*^P < .05, ^**^P < .01, ^***^P < .001 (Student’s *t*-test ).

In addition, BcHSFA4A could directly bind to the HSE elements within the promoter of *BcHSP17.6A* (Supplementary Data Fig. S5a and b). Since the *BcHSP17.6A* promoter contains two HSE motifs, these two motifs (named HSE1 and HSE2) were mutated separately (HSP M1 and M2) to confirm the most specific binding site (Table S1). The results suggested that BcHSFA4A could specifically bind to the HSE2 element in the *BcHSP17.6A* promoter (Supplementary Data Fig. S5b). Moreover, a dual LUC reporter assay also showed that, compared with the control, co-transformation of *35S:BcHSFA4A* and *proBcHSP17.6A:LUC* significantly elevated the LUC/REN ratios (Supplementary Data Fig. S5c and d), suggesting that BcHSFA4A could physically bind to the HSE motif in the *BcZAT12* and *BcHSP17.6A* promoters to increase their expression.

### The BcWRKY33A-BcHSFA4A module confers enhanced plant salt tolerance

Since BcHSFA4A interacts with BcWRKY33A and both are involved in salt tolerance, we assumed that there may be coordinated regulation between them. First, we checked whether the interaction would affect the binding ability of BcHSFA4A to its downstream genes (e.g. *BcZAT12* and *BcHSP17.6A*). EMSA results showed that the binding signals remained stable when BcHSFA4A-GST was co-incubated with the GST (Glutathione S-transferase) control or BcWRKY33A-GST (Fig. 7a; Supplementary Data Fig. S6), indicating that the binding ability of BcHSFA4A to the *BcZAT12* and *BcHSP17.6A* promoters was not affected by BcWRKY33A.

The above data suggested that BcWRKY33A has strong transcriptional activation activity, while BcHSFA4A has weak transcriptional activation activity (Figs 2c and e and 5c and d). Thus, we determined whether the interaction could affect BcHSFA4A transcriptional activation activity. As expected, by the dual LUC assay, we found that the pBD-BcHSFA4A construct slightly elevated the LUC/REN ratio (Fig. 5d and e). However, when BcHSFA4A and BcWRKY33A were co-expressed, the LUC/REN ratio was notably higher than that when only BcHSFA4A or BcWRKY33A was expressed (Fig. 5d and e), suggesting that their interaction enhances the activation ability of BcHSFA4A to regulate the expression of its downstream genes.

To further confirm this finding, we first focused on the salt stress-related genes that function downstream of *BcHSFA4A* in the WT, *35S:BcHSFA4A*, *35S:BcHSFA4A/35S:BcWRKY33A*, and *35S:BcHSFA4A/wrky33-1* lines. At the mRNA level, the expression of *ZAT12* (*AT5G59820*), *HSP17.6A* (*AT5G12030*), and *WRKY30* (*AT5G24110*) was generally higher in the *35S:BcHSFA4A/35S:BcWRKY33A* line than in the other lines (Fig. 7b) [40], suggesting that co-overexpression of *BcHSFA4A* and *BcWRKY33A* resulted in high expression of salt stress-related genes.

Then, focusing on the salt tolerance mechanisms of plants, three Na^+^/H^+^ EXCHANGERS (NHXs), NHX7/SOS1, NHX1, and NHX2, as well as HIGH-AFFINITY POTASSIUM TRANSPORTER 5 (HAK5), ARABIDOPSIS K^+^ TRANSPORTER 1 (AKT1), and CATION/H^+^ EXCHANGER 13 (CHX13), which were reported to be key regulators in maintaining Na^+^/H^+^ homeostasis by regulating the transport of various ions in the salt-responsive pathway, were selected for analysis in different lines (Fig. 7c) [4]. Among them, the expression levels of *NHX7*/*SOS1*, *HAK5*, *AKT1*, and *CHX13* in the *35S:BcHSFA4A/35S:BcWRKY33A* line were significantly higher than those in the other lines, suggesting increased salt tolerance in the *35S:BcHSFA4A/35S:BcWRKY33A* line. In addition, the expression of *NHX1* was not significantly different in various lines, and the expression of *NHX2* was lowest in the *35S:BcHSFA4A/35S:BcWRKY33A* line, indicating that the signal-response mechanism in plants under salt stress is complicated.

In addition, *ZAT12* controls genes involved in the redox process, such as ROS-scavenging ascorbate peroxidase 1 (*APX1*) [42], and negatively regulates *CBF* gene expression [43]. The results showed that *APX1* was notably induced in the *35S:BcHSFA4A/35S:BcWRKY33A* line but showed low transcript levels in the *35S:BcHSFA4A/wrky33-1* line, and *CBF*s exhibited lower levels in the *35S:BcHSFA4A/35S:BcWRKY33A* line than in the other lines (Supplementary Data Fig. S7). All these results further demonstrated that the presence or absence of BcWRKY33A affects the expression level of genes downstream of BcHSFA4A. Consistent with the gene expression results, *35S:BcHSFA4A/35S:BcWRKY33A* lines exhibited a longer root length and more lateral roots than other lines under normal conditions (Fig. 7d) and even under high salt stress (200 mM NaCl).

## Discussion

### BcWRKY33A plays a positive role in plant salt tolerance

The WRKY family contains over 100 highly divergent members that exhibit different functions in the regulation of various programs that are unique to plants [12–14]. Under salt stress, AtWRKY33 may act as a direct regulatory factor to improve plant salt tolerance. For example, recent studies found that AtWRKY33 directly binds to the *AtCYP94B1* promoter to confer root development and enhances salt tolerance in *Arabidopsis* [23]. Additionally, AtWRKY33 could directly regulate the expression of *AtKUP2*, resulting in increased root and stem length and plant survival rates [24]. Similarly, *BcWRKY33s* are differentially induced by salt stress in NHCC. Among them, *BcWRKY3A* was most obviously induced by salt stress (Fig. 1a). We speculated that BcWRKY33A, BcWRKY33B, and BcWRKY33C may be functionally redundant under salt stress, and whether their other functions differ remains to be studied.

The root system, which absorbs water and nutrition to supply the normal growth of plants, is the primary tissue that perceives salt stress from the soil [44]. Because of its importance in the plant response to salt stress, an increasing number of plant breeders have performed research on the molecular regulation process that occurs in the root system [45–48]. In rice (*Oryza sativa* L.), transgenic lines that overexpressed *OsAHL1*, *OsHAL3*, and *OsMADS25* had a larger root volume and enhanced salt tolerance under saline conditions [45–47]. In soybean (*Glycine max*), NAC (NAM, ATAF, and CUC) protein families confer salt tolerance. Overexpression of *GmNAC06* in hairy roots improves the expression of *GmUBC2* and *GmHKT1*, which could lead to more accumulation of salt-tolerant compounds and increase the salt tolerance of soybean [48]. In our study, when plants experienced salt stress, BcWRKY33A:GUS signals were extensively found in the root tissue, especially in the elongation zone and meristem zone (Fig. 1f), suggesting that BcWRKY33A mainly functions in the roots. Subsequently, we found that overexpression of *BcWRKY33A* in *A. thaliana* enhanced primary root length and lateral root density, leading to a greater growth advantage under salt stress (Fig. 3a–d). In *Stylosanthes humilis*, salt stress led to increased ABA production and decreased seed germination; however, when seeds were treated with ABA biosynthesis inhibitors, the negative effects of salt stress on seed germination were partially relieved or eliminated [36]. In rice, under salt stress conditions, ABA-responsive genes were induced and endogenous ABA accumulated; these factors inhibited the vertical elongation of the primary roots [37], which suggests that tolerance to ABA is closely related to the salt tolerance of plants. When treated with high concentrations of ABA, lines #1 and #4 exhibited a higher germination rate and longer root lengths than the WT (Fig. 3e–h), suggesting that transgenic plants harboring *BcWRKY33A* exhibit ABA tolerance. These results jointly showed that BcWRKY33A could promote seed germination, root development, and ABA tolerance, thus enhancing plant salt tolerance.

Consistent with the result that overexpression of *BcWRKY33A* could promote root development in *Arabidopsis*, *wrky33-1* mutants exhibited lateral root defects under salt treatment (Fig. 4a). Moreover, *BcWRKY33A*-silenced NHCC plants showed specific silencing of *BcWRKY33A* rather than *BcWRKY33B*/BcWRKY33C (Supplementary Data Fig. S2d–f). Under salt stress, #1, #2, and #5 also presented a salt-sensitive phenotype, decreased chlorophyll fluorescence and photosynthetic efficiency, and more severe membrane injuries and ROS accumulation (Fig. 4b–i), suggesting that impaired *BcWRKY33A* caused defects during the vegetative growth of plants. During the process of plant adaptation to salt stress, the biochemical strategies of roots include (i) selectively accumulating or eliminating ions and (ii) transporting ions into leaves. Thus, leaves are the tissues that perceive the later effects of salt stress [49], which may explain the injured leaf phenotype of *BcWRKY33A*-silenced NHCC under salt stress (Fig. 4), despite a lower GUS signal being detected in salt-treated leaves at 12 hours (Fig. 1f). Under NaCl treatment, the phenotypes of *BcWRKY33A*-overexpressing and *BcWRKY33A*-silenced plants indicated that the effect of *BcWRKY33A* was not only on root development in the seedling stage (Fig. 3) but also on vegetative growth in the development stage (Fig. 4). Similarly, *OsR3L1* separately affected root morphogenesis and vegetative traits at different stages of rice, further supporting our results [50].

In addition, BcWRKY33A shared a similar function with AtWRKY33 under biotic stress [20]. Specifically, compared with the severely compromised leaves in *wrky33-1* lines, the leaves of *35S:BcWRKY33A* lines were less damaged, indicating that BcWRKY33A also plays a positive role in defense against *B. cinerea* (Fig. 1b and c). Moreover, *BcWRKY33A* could be induced by low temperature, and its gene expression level gradually increased as the cold treatment time increased (Fig. 1e–g). However, whether BcWRKY33A is involved in the regulation of responses to low temperature or other stresses remains to be further studied.

### BcHSFA4A is involved in plant salt tolerance by directly binding to the promoters of *BcZAT12* and *BcHSP17.6A*

HSFs are well known as regulators of plant responses to severe environmental conditions [51]. In *Arabidopsis*, the overexpression of *HSFA4A* is strictly estradiol-dependent and could improve salt tolerance [29]. In our study, the overexpression line *35S:BcHSFA4A* showed more developed roots than the WT and *hsfa4a-1* mutant under salt stress (Fig. 6a; Supplementary Data Fig. S4), which also suggested the positive role of *BcHSFA4A* in salt tolerance. However, the detailed mechanism is unclear. Here, we found that *BcHSFA4A* could directly bind to the promoters of *BcZAT12* and *BcHSP17.6A* through EMSA, Y1H, and dual LUC reporter assays (Fig. 6e and f; Supplementary Data Fig. S5). In *Arabidopsis*, the transcription factor *ZAT12* could enhance the expression of ROS-scavenging genes and improve plant tolerance to salt stress [42]. SALT TOLERANCE ZINC FINGER1 (PeSTZ1) scavenges ROS accumulation by increasing the expression of the salt stress-related genes *PeZAT12* and *PeAPX2* and increasing the salt tolerance of poplar [52]. Transgenic tomato lines possessing the *BcZAT12* (*ZAT12* in *Brassica carinata*) gene showed better growth status, such as more stems, larger leaves, and longer roots and shoots than the control under the same high-salt conditions [53]. We showed that *BcZAT12* was rapidly upregulated and maintained stable high expression in roots under salt stress (Fig. 6g), which implied that *BcZAT12* is involved in the process by which plants respond to salt stress. *HSP17.6A*-overexpressing plants exhibited a high survival rate and water retention rate relative to WT, even in the absence of NaCl for 3 weeks, suggesting the better growth status of transgenic plants [54]. Thus, the role of *ZAT12* and *HSP17.6A* in plant resistance to salt stress cannot be ignored. Our findings suggested that BcHSFA4A directly binds to the promoters of *BcZAT12* and *BcHSP17.6A* to promote their expression at the transcript level (Fig. 6; Supplementary Data Fig. S5). Previously, studies on the transcriptional activation activity of HSFA4A were rather limited. Here, the weak transcriptional activation activity of BcHSFA4A implies that BcHSFA4A may cooperate with other TFs to regulate downstream genes (Fig. 5c–e).

HSFs have a conserved modular structure in which the DNA-binding domain (DBD) is always found at the N-terminal end and nuclear localization sequences (NLSs) are located downstream of the DBD [55]. The conserved structure is in agreement with our result that the N-terminus of BcHSFA4A interacts with BcWRKY33A in the nucleus (Fig. 5a and b), implying that BcHSFA4A may cooperate with BcWRKY33A to perform its functions. As homologs, BcWRKY33B and BcWRKY33C share similar domains with BcWRKY33A (Supplementary Data Fig. S1a and b), and we speculate that BcWRKY33B and BcWRKY33C also interact with BcHSFA4A, which requires further analysis. In addition, the transcriptional activity of WRKY33 depends on mitogen-activated protein kinase MPK3/MPK6-mediated phosphorylation modifications [56, 57]. At the same time, HSFA4A, as a substrate, interacts with MPK3 and MPK6 in yeast and plant cells [29]. Hence, our result that BcWRKY33A interacts with BcHSFA4A may provide a new perspective for understanding the relationship among WRKY33, HSFA4A, and the protein kinases MPK3 and MPK6.

### Coordinated regulation of BcWRKY33A and BcHSFA4A confers plant salt tolerance

Previous studies have shown that most proteins need molecular chaperones to perform their functions under stress conditions [58]. For instance, SIB1 and SIB2 could interact with WRKY33 to ensure that WRKY33 functions in plant defense [21]. The latest research showed that WRKY33 and WRKY12 cooperatively control the expression of *RAP2.2* to modulate the submergence response [39], suggesting that protein–protein interactions regulate downstream genes in different ways to resist abiotic stress. However, the detailed regulatory mechanisms of WRKY33 with regard to its interacting proteins still need further exploration. In our study, BcWRKY33A, which physically interacts with BcHSFA4A (Fig. 5a and b), was identified as the regulatory partner of BcHSFA4A regarding downstream genes (Figs 2c, e and f and 5d and e).

Here, *BcHSFA4A* was not affected by *BcWRKY33* at the transcript level (Supplementary Data Figs S2c and d and S3b and c), suggesting that their physical interactions may function at the protein level. Next, we hypothesized that the interaction between BcWRKY33A and BcHSFA4A may affect the binding ability or transcriptional activation activity of BcHSFA4A to its downstream genes. EMSA experiments showed that the presence or absence of BcWRKY33A did not affect the binding ability of BcHSFA4A to the promoters of *BcZAT12* and *BcHSP17.6A* (Fig. 7a; Supplementary Data Fig. S6). However, the presence of BcWRKY33A could significantly improve the transcriptional activation activity of BcHSFA4A (Fig. 5d and e); in other words, the interaction between BcWRKY33A and BcHSFA4A allows BcHSFA4A to significantly activate the expression of *BcZAT12* and *BcHSP17.6A*. Similarly, WRKY33 can physically bind to specific motifs/elements in the promoters of defense-related genes; however, only WRKY33 phosphorylated by MPK3/6 under phytopathogen attack could activate the expression of its downstream genes to improve plant defense [56, 59]. This activation of WRKY33 might be accompanied by posttranscriptional protein modification and other unknown regulatory mechanisms [39], and we speculated that the synergistic enhancement of *BcZAT12* and *BcHSP17.6A* by BcWRKY33A and BcHSFA4A likely relies on the same or a similar modification, which needs to be further studied.

The synergistic regulatory role of BcWRKY33A and BcHSFA4A under salt stress was also confirmed, as shown in Fig. 7b. Co-overexpression of *BcWRKY33A* and *BcHSFA4A* in *Arabidopsis* significantly upregulated the expression levels of genes downstream of *BcHSFA4A*, such as *ZAT12* [42], *HSP17.6A* [54], and *WRKY30* [40] (Fig. 7b). Focusing on the salt stress signaling pathway, NHX7/SOS1 mainly transports sodium ions and regulates the exclusion of sodium ions in root tissue [4]. Under salt stress conditions, members of the potassium transporters, such as *HAK5* and *AKT1*, could facilitate potassium transport [60, 61], while *CHX13* mainly functions in root growth direction on a salt gradient [62]. Our data showed that the expression levels of these members in various lines differed, indicating the enhanced tolerance of *35S:BcHSFA4A/35S:BcWRKY33A* lines (Fig. 7c). However, the biological roles of these transporters in different lines remain to be studied. Moreover, *35S:BcHSFA4A/35S:BcWRKY33A* lines exhibited a greater growth advantage than *35S:BcHSFA4A* and *35S:BcWRKY33A* lines under both normal and salt stress conditions (Fig. 7d). These findings further support the important role of the BcWRKY33A-BcHSFA4A module in the response to salt stress.

**Figure 8:**
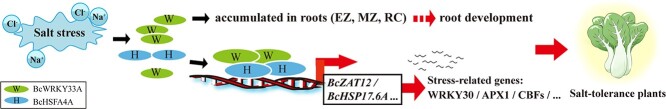
A proposed working model of how BcWRKY33A-BcHSFA4A improves salt tolerance in NHCC. Under salt stress, BcWRKY33A is markedly induced and accumulates in the roots, especially in the elongation zone (EZ), meristem zone (MZ), and root cap (RC), to promote root development via an unknown mechanism. The abundant BcWRKY33A protein, which physically interacts with BcHSFA4A, can accurately and efficiently regulate downstream salt stress-related genes by forming the BcWRKY33A-BcHSFA4A module, thereby promoting salt tolerance in plants.

At the same time, salt stress-related genes, such as *APX1* and *CBF*, which are positively or negatively regulated by ZAT12 [42, 43], were more noticeably regulated in *35S:BcHSFA4A/wrky33-1* and *35S:BcHSFA4A/35S:BcWRKY33A* lines*,* respectively (Supplementary Data Fig. S7). It should be noted that previous studies suggested that *cbf* triple mutants had fewer lateral roots and a lower seedling fresh weight than the WT under salt stress [63]. However, we showed that the *35S:BcHSFA4A/35S:BcWRKY33A* lines, which exhibited increased salt tolerance, showed diverse expression level changes of *CBF*s, implying that the mechanism of *CBF* gene regulation of salt stress is complex. Collectively, our data suggest that BcWRKY33A directly interacts with BcHSFA4A to form a regulatory module that positively controls downstream stress-related genes and ultimately improves the salt tolerance of plants (Fig. 8).

## Materials and methods

### Plant materials and growth conditions

The Colombia-0 (Col-0) ecotype of the *A. thaliana* was selected as a control in some experiments. The T-DNA insertional mutants *wrky33-1* (SALK_006603) and *hsfa4a-1* (SALK_036303C) were obtained from the Arabidopsis Biological Resource Center (ABRC). Homozygous *wrky33-1* and *hsfa4a-1* mutant plants were identified by PCR according to previous studies [20, 64] (Supplementary Data Table S1). To generate stable *BcWRKY33A* and *BcHSFA4A* transgenic plants, the coding regions of *BcWRKY33A* and *BcHSFA4A* were amplified from cDNA of the NHCC cultivar ‘Suzhouqing’ using the primers BcWRKY33A-F/-R and BcHSFA4A-F/-R, respectively (Supplementary Data Table S1). *35S:BcWRKY33A-GFP* was constructed using Gateway technology as described in a previous study [65]. *35S:BcHSFA4A-FLAG* was constructed by the homologous recombination method using the CloneUFO™ One Step Cloning Kit (C101, ATG Biotechnology, China) [66]. The *35S:BcHSFA4A-FLAG* construct was transformed into genetically stable lines with *35S:BcWRKY33A-GFP* and *wrky33-1* backgrounds to obtain *35S:BcHSFA4A/35S:BcWRKY33A* and *35S:BcHSFA4A/wrky33-1* lines, respectively. All transgenic materials in this study were generated by *Agrobacterium*-mediated transformation and the floral dip method [67], and genetically stable *T*_3_ lines were used for analysis. NHCC cultivar ‘Suzhouqing’, WT and transgenic *Arabidopsis*, and *Nicotiana benthamiana* (tobacco) plants were grown at 23°C in a climate chamber with long-day conditions (16/8 hours light/dark, 250 μmol m^−2^ s^−1^) at Nanjing Agricultural University (Nanjing, China). All primers used for vector construction are shown in Supplementary Data Table S1.

### Bioinformatics analysis

The conserved domains of the BcWRKY33A and BcHSFA4A proteins were analyzed using the SMART online tool (http://smart.embl.de/). The WRKY33 protein sequences from various species were collected from the NCBI database (https://www.ncbi.nlm.nih.gov/), the multiple sequence alignments of the amino acid sequences of WRKY33 from various species were analyzed by Jalview software [68], and the phylogeny was derived by MEGA X software with the neighbor-joining algorithm and 1000 bootstrap replications [69]. All of the gene sequences in this study were obtained from the NHCC genome (*B. rapa* NHCC001, ‘Suzhouqing’) [30].

### Quantitative real-time PCR

Total RNA was extracted by using a FastPure Plant Total RNA Isolation Kit (Vazyme Biotech Co., Ltd, China), and cDNA was synthesized using an *Evo M-MLV* Mix Kit with gDNA Clean for qPCR [Accurate Biotechnology (Hunan) Co., Ltd, China]. Then, the samples were analyzed by qPCR using Hieff^®^ qPCR SYBR Green Master Mix (High Rox Plus) [Yeasen Biotechnology (Shanghai) Co., Ltd, China] according to the manufacturer’s instructions on a StepOnePlus system (Applied Biosystems, USA). The relative expression of genes was analyzed by the 2^-ΔΔCT^ method and normalized to *ELF4A* (AT1G80000) and *BcPP2A* (BraC07g034860.1) for *Arabidopsis* and NHCC, respectively [70, 71]. All primers used for qPCR analysis were designed with the Primer-BLAST tool (https://www.ncbi.nlm.nih.gov/tools/primer-blast/), as shown in Supplementary Data Table S1.

### β-Glucuronidase staining and expression

The 5′ upstream region of the *BcWRKY33A* sequence (−2000 to −1; the translation initiation site was counted as +1) was amplified from the genomic DNA of NHCC cultivar ‘Suzhouqing’ and inserted into the pFAST-G04 vector by the Gateway system [65]. Twelve-day-old *BcWRKY33A:GUS* transgenic seedlings were placed on MS medium with mock, salt (100 mM NaCl, 12 hours), or cold (4°C, 12 hours) treatment, and the histochemical localization of GUS was analyzed as described by Kim *et al*. [72]. Stained tissues were photographed by digital video microscopy (DVM6a, Leica, Germany). Moreover, the *GUS* gene expression levels were also measured by qPCR across the different treatments. Primers for the relative expression of *GUS* analysis (qGUS-F/R) are shown in Supplementary Data Table S1.

### Subcellular localization analysis

The *BcWRKY33A* coding sequence without a termination codon was cloned and fused with green fluorescent protein (GFP) in the vector pEarlygate103. The *35S:BcWRKY33A-GFP* construct and the histone H2B-RFP fusion were individually transformed into GV3101 [33] and subsequently infiltrated into 35-day-old tobacco seedlings. After 48–96 hours, the different fluorescent signals were inspected via a confocal laser scanning microscope (LSM780, Zeiss, Germany) [73].

### Dual-luciferase reporter assay

For transcriptional activation analysis *in vivo* [34], the coding sequence of *BcWRKY33A* or *BcHSFA4A* was fused with the constructed pBD vector driven by the 35S promoter as the effector. The recombined effectors and double reporter vector (GAL4-LUC) were subsequently transferred into GV3101 and then co-infiltrated into tobacco leaves [34]. d-Luciferin was applied on the adaxial side of the leaves, followed by LUC imaging using a plant imaging system (Night Shade LB 985, Berthold). LUC and REN luciferase activities were measured using a Dual-Luciferase Assay Kit (Promega) on a Cytation3 Plate Reader (BioTek). The LUC/REN values reflect the transcriptional activation activity.

To assay the binding activity of BcHSFA4A to the promoter of *BcHSP17.6A*, the promoter sequence (570 bp upstream of the initiation codon) of *BcHSP17.6A* was selected and inserted into the reporter vector pGreenII 0800-LUC, and *35S:BcHSFA4A-FLAG* was used as the effector. *35S:FLAG* and the promoter sequence (1450 bp upstream of the initiation codon) of *BcCYP71A13* (BraC03g016690.1), which has no HSE motifs, were used as the negative controls. The constructed effector and reporter plasmids were co-infiltrated into tobacco leaves. The LUC/REN values reflect the binding activity. All primers used in the dual-luciferase reporter assay are listed in Supplementary Data Table S1.

### Virus-induced gene silencing

The BcWRKY33A-silenced plants were generated by Yu’s method, as previously described [38]. Briefly, an interfering 40-bp fragment (5′-ATGAATGGTTCTGTTAATTGGTCACAACAAACCGCAAGAGCTCTTGCGGTTTGTTGTGACCAATTAACAGAACCATTCAT-3′) of the *BcWRKY33A* coding sequence (underlined) and its antisense sequence were inserted into the pTY vector [38]. NHCC ‘Suzhouqing’ seedlings were bombarded with the constructed vector using a gene gun (Biolistic PDS-1000/He, Bio–Rad) [74]. The empty pTY vector was introduced into seedlings to obtain control plants. Seven to 10 days after infection, new leaves from seedlings were collected to verify the expression of *BcWRKY33A*. After qPCR, we selected three pTY-BcWRKY33A lines (#1, #2, and #5) that showed 60–70% repression of *BcWRKY33A* transcript levels in the silenced plants relative to the pTY control for further analysis.

### Physiological analysis and histochemical staining

To record the chlorophyll fluorescence *F*_v_/*F*_m_ ratios, a chlorophyll fluorimeter (IMAGING-PAM, Walz, Germany) was used. Electrolyte leakage was measured by Dahro’s method [75], and MDA contents and superoxide dismutase (SOD) and catalase (CAT) activity were measured using commercial kits (S0131S, Beyotime, China; BC0175, BC0205, Solarbio, China). The total protein content of the samples was measured using a Bradford Protein Assay Kit (T9310A, Takara, Japan). Histochemical staining with 3,3′-diaminobenzidine (DAB) and nitroblue tetrazolium (NBT) was performed as reported previously to measure the accumulation of H_2_O_2_ and O_2_^.−^ in NHCC leaves [76].

### Y2H and Y1H assays

To identify the proteins that interact with BcWRKY33A, a library screening assay was performed using the Matchmaker™ GoldYeast Two-Hybrid System (Clontech). To select the appropriate bait for two-hybrid screening, a bait self-activation test was performed on BcWRKY33A. Then, cBcWRKY33A containing two conserved WRKY domains was selected as bait and cloned into the vector pGBKT7, and the NHCC library was used as prey and cloned into the vector pGADT7. Screening of the Y2H library was performed according to the manufacturer’s protocol.

For interaction verification, the coding sequences of *cBcWRYY33A* and *BcHSFA4A* were recombined into the pGBKT7 and pGADT7 vectors to fuse with the binding domain (BD) and activation domain (AD), respectively, to create the bait and prey. The constructs were co-transformed into the yeast strain Y2H Gold and then cultured on DDO/X medium (SD/−Leu−Trp) containing 4 mg mL^−1^ X-α-Gal at 30°C for 3 days. Positively transformed clones were plated on QDO/X medium (SD/−Trp/−His/−Leu/−Ade) containing 4 mg mL^−1^ X-α-Gal to test the protein interaction.

For transcriptional activation analysis in yeast cells [34], full-length BcWRKY33A, the N-terminus of BcWRKY33A [1–158 amino acids (aa), nBcWRKY33A], the C-terminus of BcWRKY33A (159–476 aa, cBcWRKY33A) and full-length BcHSFA4A were amplified by PCR and ligated into the pGBKT7 vector. The recombinant plasmids were transformed into Y2H Gold and then plated on SDO (SD/−Trp), DDO (SD/−Trp/−His) and QDO (SD/−Trp/−His/−Leu/−Ade) dropout media with or without X-α-Gal (Clontech, Cat. 630 462) and incubated at 30°C for 3 days to test their transcriptional activation activity.

To check the binding ability of BcHSFA4A to the *BcZAT12* promoter, the yeast one-hybrid assay (Y1H) assay was performed using the Matchmaker™ GoldYeast One-Hybrid System (Clontech). Briefly, a series of truncated *BcZAT12* promoter fragments containing different HSE elements were amplified from the genomic DNA of NHCC cultivar ‘Suzhouqing’. The amplified DNA fragments were inserted in front of the *AUR1-C* gene, which is a resistance gene in the pAbAi plasmid that confers resistance to aureobasidin A (AbA), to obtain the bait plasmid pAbAi-BcZAT12-HSEs. Then, bait reporter strains were generated by integrating linearized pAbAi-BcZAT12-HSEs plasmids into the yeast strain Y1H Gold using a commercial kit by the lithium acetate method (Clontech, Cat. 630439). The plasmid pGADT7-BcHSFA4A and empty plasmid pGADT7 were transformed into bait reporter strains, and then the appropriate inhibition concentration of AbA was added to SD/−Ura/−Leu medium at 30°C for 3 days for protein–DNA interaction validation.

### Bimolecular fluorescence complementation analysis

The full-length coding sequences of *BcWRKY33A* and *BcHSFA4A*, without stop codons, were inserted into the pFGC-YN173 and YC155 (YN and YC) vectors, respectively [77]. In addition, to map the interaction domains, the N-terminal of BcHSFA4A (aa 1–121, BcHSFA4A-N), the middle fragment of BcHSFA4A (aa 122–252, BcHSFA4A-M), and the C-terminal of BcHSFA4A (aa 253–389, BcHSFA4A-C) were inserted into the YC vector to verify their possible interaction with BcWRKY33A. The resulting constructs were transferred into GV3101 and subsequently used to transform 5-week-old *N. benthamiana* leaves [78]. After 48–96 hours, yellow fluorescent protein (YFP) was visualized via a confocal laser scanning microscope (LSM780, Zeiss, Germany) [73].

### Electrophoretic mobility shift assay

The *BcWRKY33A* and *BcHSFA4A* coding sequences were cloned into the pGEX-4 T-1 vector and transferred into the *Escherichia coli* strains Rosetta (DE3) and BL21 (DE3), respectively. After induction by 1 mM isopropyl β-d-thiogalactoside (IPTG) for 12 hours at 28°C, the fusion protein was purified using GST-Sefinose Resin 4FF (Settled Resin) (Sangon Biotech, China). The EMSA experiment was performed using a Chemiluminescent EMSA Kit (GS009, Beyotime, Shanghai, China). In brief, bound and unbound DNA–protein complexes were separated by 6% native polypropylene gels, and different signal bands were transferred to nylon membranes by electrophoresis (Biosharp, China) and then subjected to UV crosslinking and chemiluminescence detection (ChemiDoc MP, USA).

## Acknowledgements

This study was supported by National Natural Science Foundation of China (32072575), the National Vegetable Industry Technology System (CARS-23-A16), Jiangsu province’s seed industry revitalization project (JBGS[2021]015) and the Priority Academic Program Development of Jiangsu Higher Education Institutions (PAPD). We thank anyone who helped us in this study, including undergraduate student Yanwen Zhang, who helped us draw the cartoon NHCC in Fig. 8.

## Author contributions

L.T.K. designed the study. W.H.Y. and L.Z.B. conducted the experiments, analyzed the data, and wrote the manuscript. L.T.K. and H.X.L. revised the manuscript. R.H.B., Z.C.W., X.D., and L.Y. helped prepare the samples. All authors read and approved the final manuscript.

## Data availability

Some or all data generated or used during the study are available from the corresponding author on reasonable request.

## Conflict of interest

The authors declare no conflict of interest.

## Supplementary data

Supplementary data is available at *Horticulture Research* online.

## Supplementary Material

Web_Material_uhac113
