# Correction to: Regulatory interaction of BcWRKY33A and BcHSFA4A promotes salt tolerance in non-heading Chinese cabbage [*Brassica campestris* (syn. *Brassica rapa*) ssp. *chinensis*]

**DOI:** 10.1093/hr/uhae207

**Published:** 2024-08-10

This is a correction to: Huiyu Wang, Zhubo Li, Haibo Ren, Changwei Zhang, Dong Xiao, Ying Li, Xilin Hou, Tongkun Liu, Regulatory interaction of BcWRKY33A and BcHSFA4A promotes salt tolerance in non-heading Chinese cabbage [*Brassica campestris* (syn. *Brassica rapa*) ssp. *chinensis*], *Horticulture Research*, Volume 9, 2022, https://doi.org/10.1093/hr/uhac113

The original version of this article contained an error in Fig. 2c and a wrong description in the **Results** section, in which two labels left are in reverse order in Fig. 2c, and the corresponding explain of Fig. 2C is also mistaken. The correct version is:


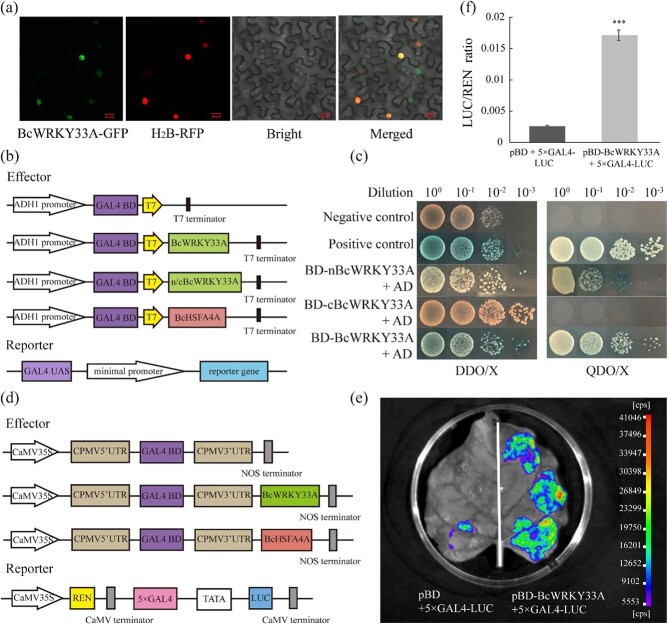


The results indicated that nBcWRKY33A had strong transcriptional activation activity, although it was slightly weaker than that of full-length BcWRKY33A (Fig. 2c). This replaces the previous incorrect version:


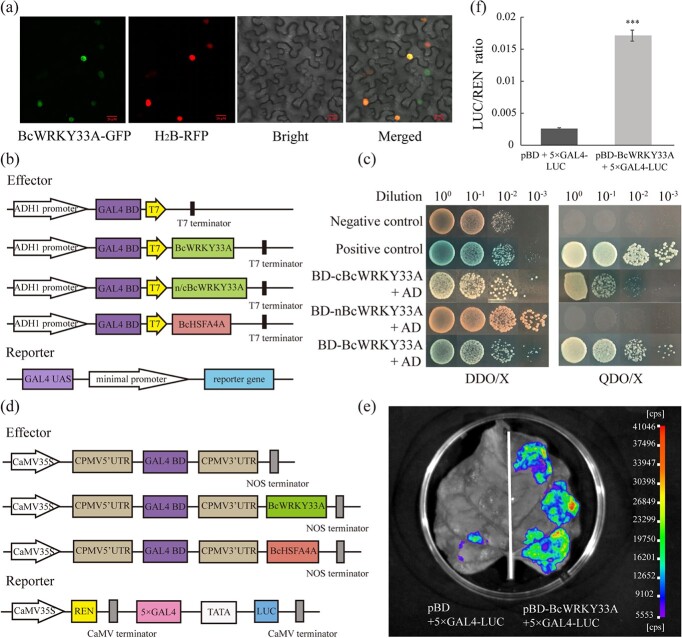


The results indicated that cBcWRKY33A had strong transcriptional activation activity, although it was slightly weaker than that of full-length BcWRKY33A (Fig. 2c).

The original version of this article contained an error in Fig. 3e, in which the picture of line # 4 at 0 μM ABA concentration was incorrectly used. The correct version is: 


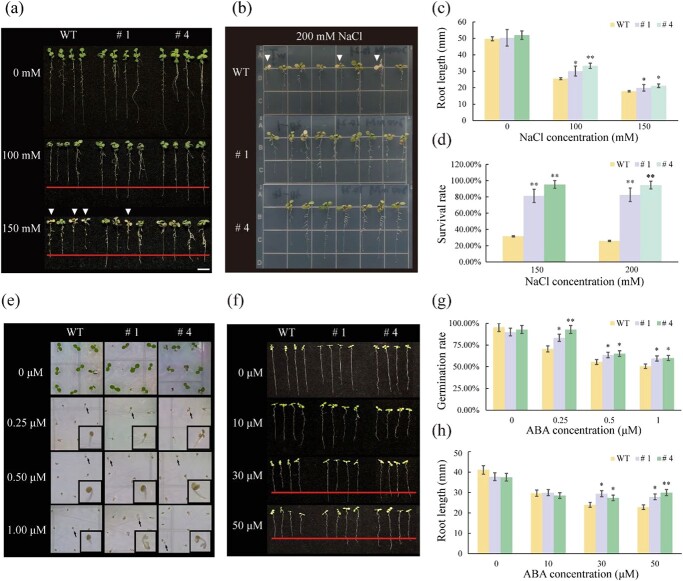


which replaces the previous incorrect version:


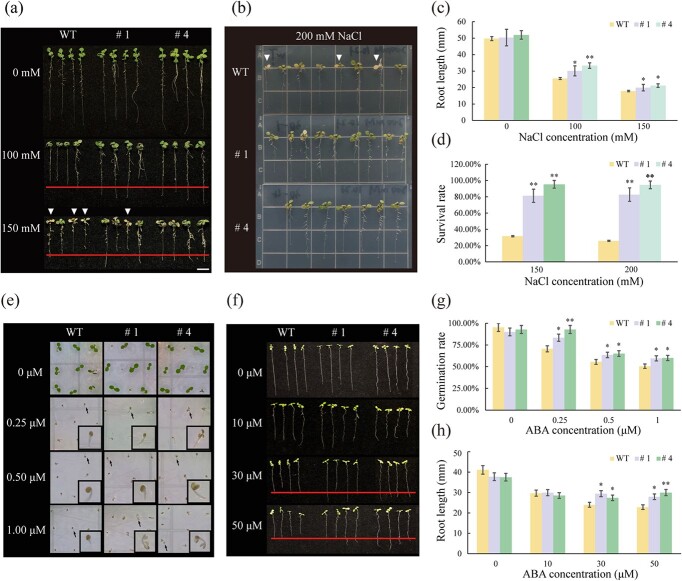


These errors and emendations have been outlined only in this correction notice to preserve the version of record.

