# Supplementary material for: Regulatory interaction of BcWRKY33A and BcHSFA4A promotes salt tolerance in non-heading Chinese cabbage [Brassica campestris (syn. Brassica rapa) ssp. chinensis]
Source: Hortic Res. 2022 May 17;9:uhac113. doi: 10.1093/hr/uhac113 (PMC9273956; doi:10.1093/hr/uhac113)
Supplement: Web_Material_uhac113 [file web_material_uhac113.zip › 4.21-Suppelement Figures.docx]

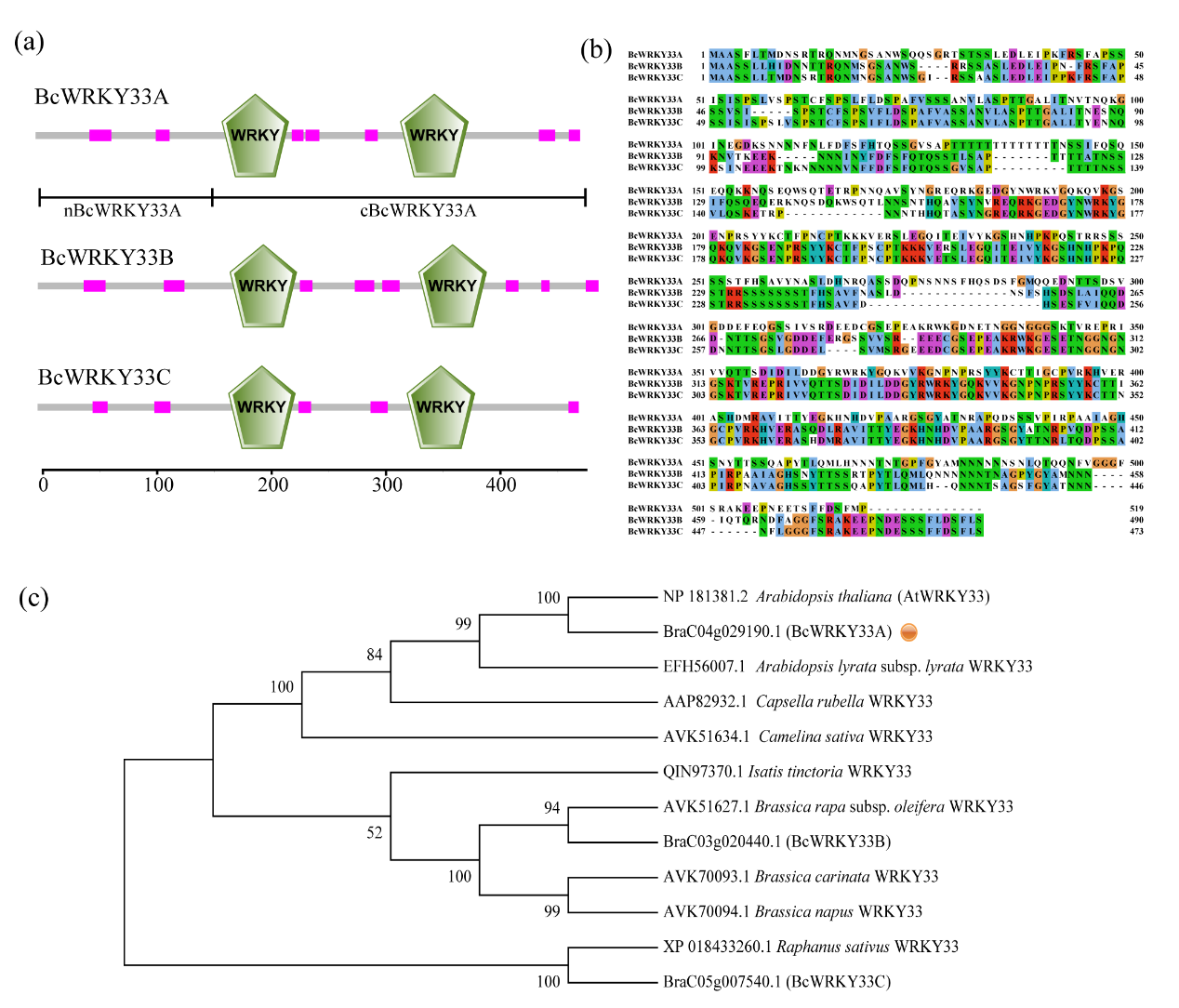


**Fig. S1: Identification of BcWRKY33s.**

**a** A schematic illustration of the domain organization of BcWRKY33s. Green polygons represent conserved WRKY domains. The small squares represent low complexity region. The nBcWRKY33A means the N-terminal of BcWRKY33A without WRKY domain (1-158 aa), and the cBcWRKY33A means the C-terminal of BcWRKY33A containing two WRKY domains (159-478 aa). **b** Multiple sequence alignments of the amino acid sequences of BcWRKY33s in NHCC. **c** Phylogenetic tree of BcWRKY33s and its homologs in various species. The neighbor-joining method was used and the bootstrap value was set 1000 to construct a phylogenetic tree.


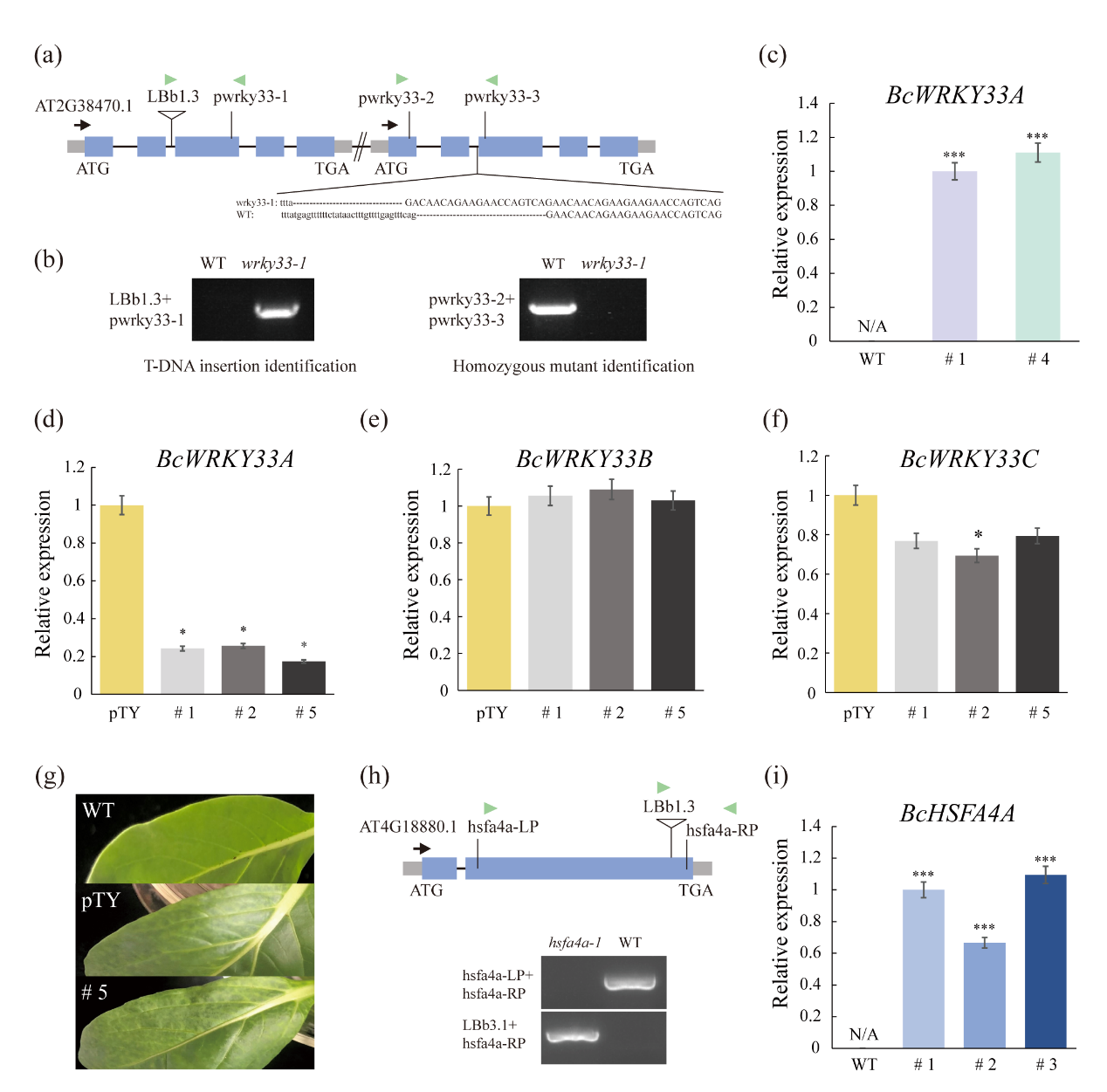


**Fig. S2: Identification of T-DNA insertion mutants, overexpressing transgenic lines and NHCC silencing lines.**

**a** The identification of the T-DNA insertion mutant SALK_006603. LBb1.3 primer is obtained from the website (http://signal.salk.edu/tdnaprimers.2.html). LBb1.3, pwrky33-1, -2, -3 primers were used for genotyping according to the ‘plant materials’ section in manuscript. The position of the T-DNA insertion, the primers and genomic rearrangements deduced from the sequencing of the mutant alleles are shown. The black arrows show the direction of transcription. Green arrows represent primers in different directions. **b** Genotyping of the T-DNA insertion mutant (left) and homozygous mutant (right) of *wrky33-1*. **c** Expression level of *BcWRKY33A* in three-week old WT and transgenic *Arabidopsis* lines (# 1, # 4). N/A represents no expression. **d-f** Expression level of *BcWRKY33A* (**d**), *BcWRKY33B* (**e**), *BcWRKY33C* (**f**) in six-week-old pTY and BcWRKY33A-silenced NHCC lines (# 1, # 2, # 5). **g** The viral infection mosaic phenotype of in six-week-old WT, pTY and BcWRKY33A-silenced NHCC lines (# 5). **h** The identification and genotyping of the T-DNA insertion mutant SALK_036303C. LBb1.3, hsfa4a-LP, -RP primers are obtained from the website (http://signal.salk.edu/tdnaprimers.2.html). **i** Expression level of *BcHSFA4A* in three-week old WT and transgenic *Arabidopsis* lines (# 1, # 2, # 3). N/A represents no expression. All data are averages of three independent experiments, and error bars represent SEM. **P* < 0.05 and ****P* < 0.001 (Student’s *t* test).


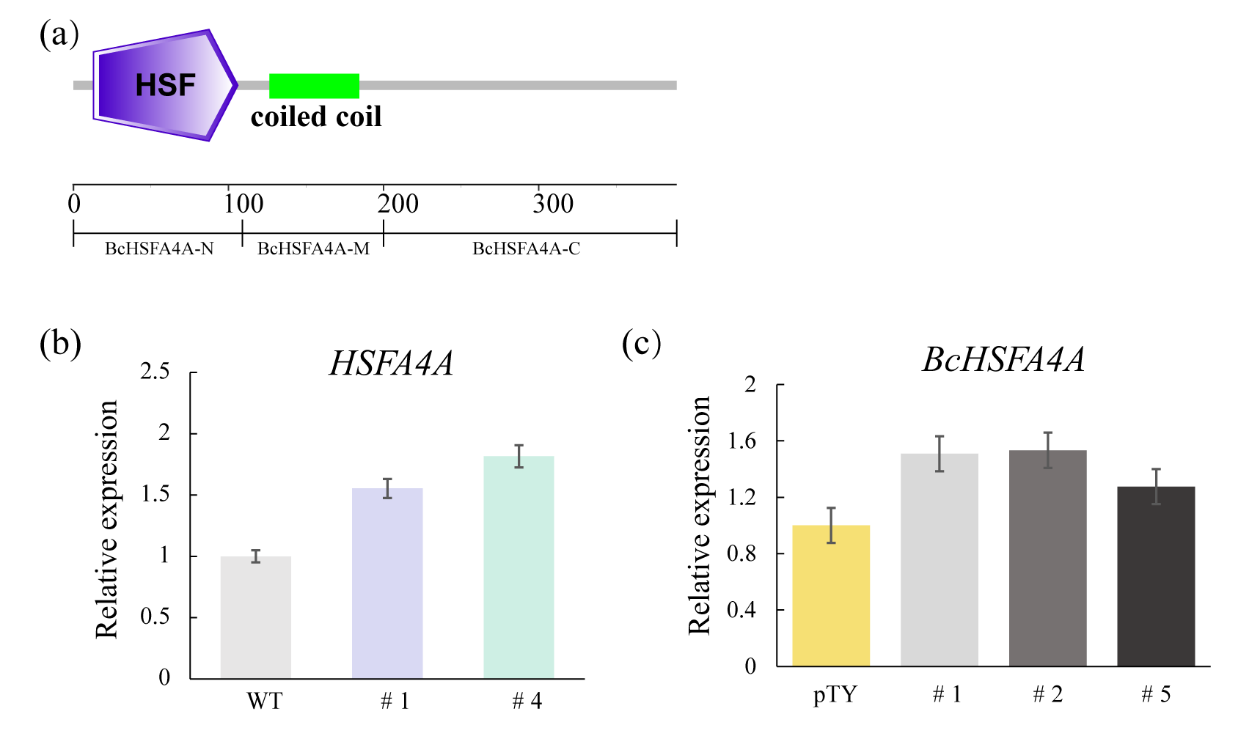


**Fig. S3: Analysis of BcHSFA4A and HSFA4A.**

**a** The schematic illustration of BcHSFA4A conserved domain. Purple polygons represent conserved HSF domains. The green rectangle represents coiled-coil region. BcHSFA4A-N means the N-terminal fragment of BcHSFA4A containing a conserved HSF domain (amino acids 1-121), BcHSFA4A-M means the middle fragment of BcHSFA4A containing a coiled-coil region (amino acids 122-252), and BcHSFA4A-C means the C-terminal fragment of BcHSFA4A (amino acids 253-389). **b** Expression level of *HSFA4A* in three-week old WT and transgenic Arabidopsis lines (# 1, # 4). **c** Expression level of *BcHSFA4A* in six-week-old pTY and BcWRKY33A-silenced NHCC lines (# 1, # 2, # 5).


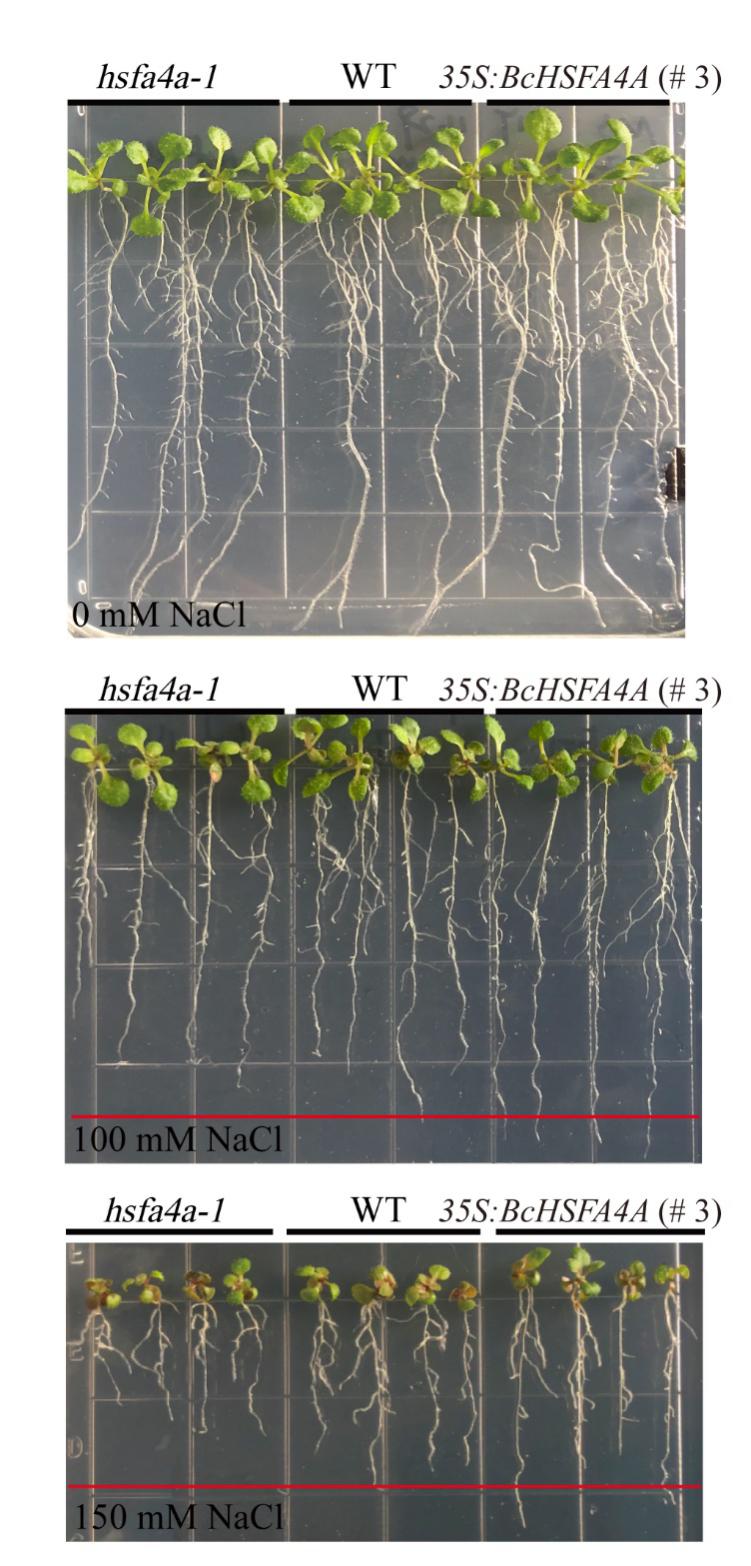


**Fig. S4****: The phenotype of *hsfa4a-1*, WT and *35S:BcHSFA4A* under normal condition and salt treatment.**

The seedlings were grown on the medium with different concentration of NaCl (0, 100, 150 mM) for 10 days. The red lines represent the longest root length of the WT.


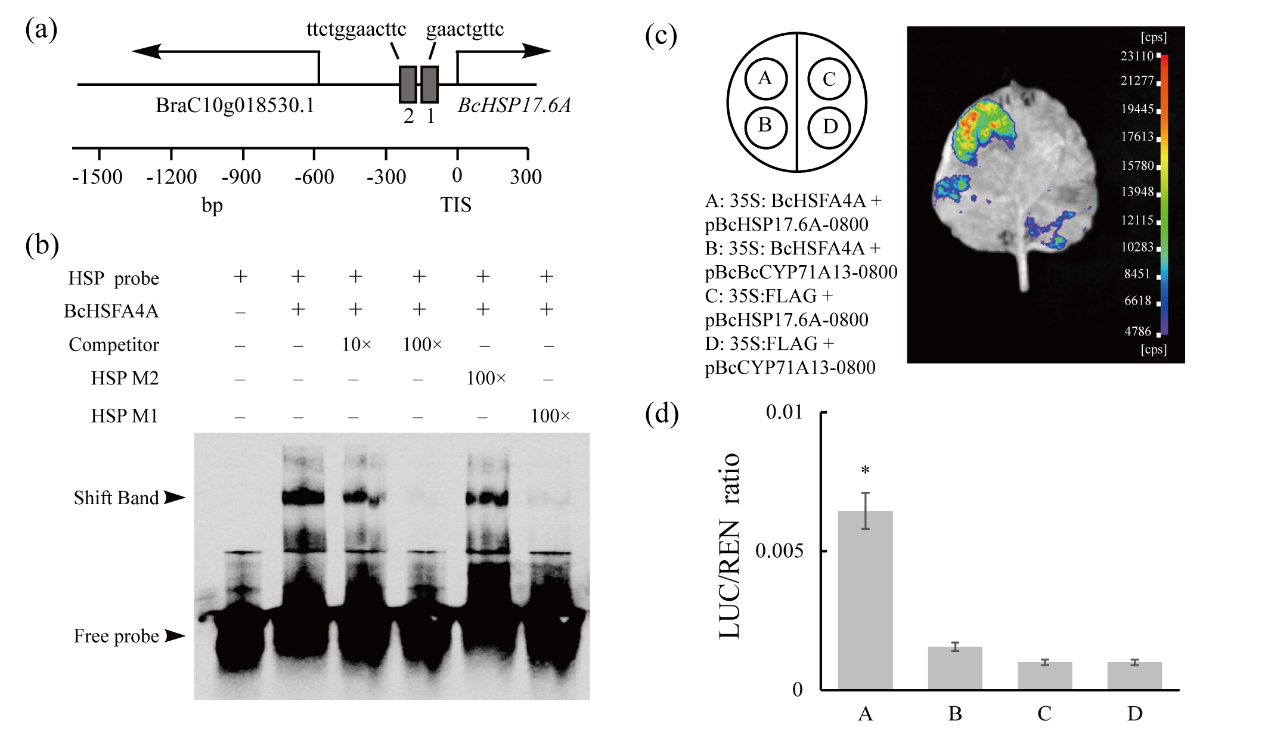


**Fig. S5: BcHSFA4A directly binds to the HSE motif in the *BcHSP17.6A* promoter.**

**a** The distribution of HSE motifs in promoter of *BcHSP17.6A*. HSE motifs are indicated by grey boxes. The sequences of two HSE motifs in promoter of *BcHSP17.6A* are shown above the target region. Arrow indicates translation initiation site (TIS). **b** EMSA was used to detect the direct and specific binding of BcHSFA4A to the *BcHSP17.6A* promoter. EMSA of the biotin-labeled oligonucleotide derived from the putative binding site of *BcHSP17.6A* promoter in the presence or absence of cold competitor and mutated probe (HSP M1 and HSP M2). Purified BcHSFA4A protein (4 μg) was incubated with 50 nM biotin-labeled probes. For the competition test, cold competitor and mutated probes at 10- (10x) or 100-fold (100x) concentrations were added in the experiment. Presence (+) or absence (-) of the components are shown on the top. **c** Imaging of LUC activity in tobacco leaves injected with different constructs. **d** The LUC activities were measured by the ratio of LUC/REN in tobacco leaves. A-F corresponds to the label in (c). All data are averages of three independent experiments, and error bars represent SEM. **P* < 0.05, (Student’s *t*-test).


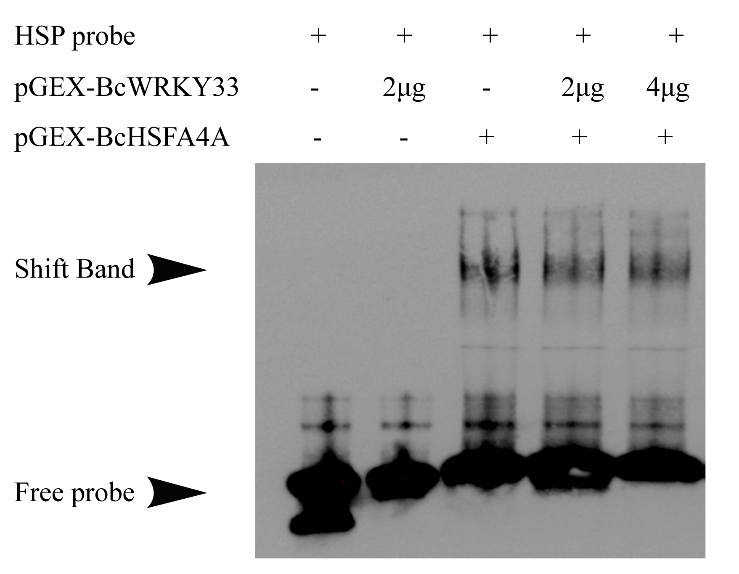


**Fig. S6: The binding ability of BcHSFA4A to *BcHSP17.6A* promoter is not affected by BcWRKY33A.**

EMSA was used to detect the effect of BcWRKY33A on the binding ability of BcHSFA4A to the *BcHSP17.6A* promoter. Presence (+) or absence (-) of the components are shown on the top.


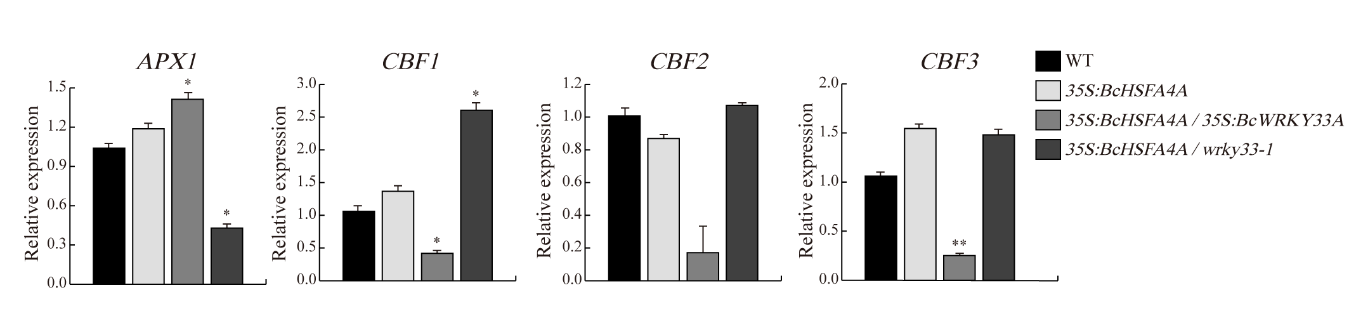
**Fig. S7:** **The expression levels of downstream genes of *BcZAT12* in different lines.**

The expression levels of ROS scavenging ascorbate peroxidase 1 (*APX1*) and three *CBF* genes (*CBF1*, *CBF2* and *CBF3*) in WT, *35S:BcHSFA4A*, *35S:BcHSFA4A / 35S:BcWRKY33A*, and *35S:BcHSFA4A / wrky33-1* lines. All data are averages of three independent experiments, and error bars represent SEM. **P* < 0.05, ***P* < 0.01 (Student’s *t*-test).
